# Identification of distinct capsule types associated with *Serratia marcescens* infection isolates

**DOI:** 10.1371/journal.ppat.1010423

**Published:** 2022-03-30

**Authors:** Mark T. Anderson, Stephanie D. Himpsl, Lindsay A. Mitchell, Leandra G. Kingsley, Elizabeth P. Snider, Harry L. T. Mobley

**Affiliations:** University of Michigan, Michigan Medicine, Department of Microbiology and Immunology, Ann Arbor, Michigan United States of America; Stanford University School of Medicine, UNITED STATES

## Abstract

*Serratia marcescens* is a versatile opportunistic pathogen that can cause a variety of infections, including bacteremia. Our previous work established that the capsule polysaccharide (CPS) biosynthesis and translocation locus contributes to the survival of *S*. *marcescens* in a murine model of bacteremia and in human serum. In this study, we determined the degree of capsule genetic diversity among *S*. *marcescens* isolates. Capsule loci (KL) were extracted from >300 *S*. *marcescens* genome sequences and compared. A phylogenetic comparison of KL sequences demonstrated a substantial level of KL diversity within *S*. *marcescens* as a species and a strong delineation between KL sequences originating from infection isolates versus environmental isolates. Strains from five of the identified KL types were selected for further study and electrophoretic analysis of purified CPS indicated the production of distinct glycans. Polysaccharide composition analysis confirmed this observation and identified the constituent monosaccharides for each strain. Two predominant infection-associated clades, designated KL1 and KL2, emerged from the capsule phylogeny. Bacteremia strains from KL1 and KL2 were determined to produce ketodeoxynonulonic acid and *N*-acetylneuraminic acid, two sialic acids that were not found in strains from other clades. Further investigation of KL1 and KL2 sequences identified two genes, designated *neuA* and *neuB*, that were hypothesized to encode sialic acid biosynthesis functions. Disruption of *neuB* in a KL1 isolate resulted in the loss of sialic acid and CPS production. The absence of sialic acid and CPS production also led to increased susceptibility to internalization by a human monocytic cell line, demonstrating that *S*. *marcescens* phagocytosis resistance requires CPS. Together, these results establish the capsule genetic repertoire of *S*. *marcescens* and identify infection-associated clades with sialic acid CPS components.

## Author summary

*Serratia marcescens* is a bacterial species with a broad natural distribution that is capable of causing infections, particularly in individuals with underlying health conditions. One consideration for organisms, such as *S*. *marcescens*, that can thrive in diverse environments is whether specific genetic lineages are better suited to causing infection than others. The capsular polysaccharide of *S*. *marcescens* is a variable surface structure that is important for bacterial survival in an experimental model of bloodstream infection and we sought to determine the spectrum of genetic diversity within the capsule-encoding genetic region among both infection and environmental isolates. Here, we demonstrate that *S*. *marcescens* strains originating from infection and environmental sources segregate into distinct clades based on capsule gene phylogeny. The majority of infection isolates analyzed were further differentiated into two types. Strains from both of the primary infection-associated capsule types produce sialic acids, a previously unrecognized trait that is likely to have implications in capsule polysaccharide function and host immune system recognition.

## Introduction

*Serratia marcescens* is an opportunistic pathogen that causes infections of the urinary tract, respiratory system, and bloodstream. *S*. *marcescens* is also a problematic healthcare-associated pathogen. In a recent epidemiological survey spanning several countries, *Serratia* species were responsible for 3.4% of bloodstream infections (BSI) and 5.3% of pneumonias resulting from admission to an intensive care unit [1], making it among the ten most frequently observed etiologic agents of these healthcare-associated infections. *S*. *marcescens* is also among the ten most common microbial agents of all BSI [2,3]. BSI caused by *S*. *marcescens* are particularly concerning among pediatric populations, as rates of infection have increased over the past decade and are associated with worse outcomes compared to non-*Serratia* BSI [4]. In addition to its role as a human pathogen, *S*. *marcescens* has a strikingly wide natural distribution. *S*. *marcescens* can be isolated from numerous environmental niches, including soil and water, and is found in both pathogenic and non-pathogenic relationships with plants, animals, and insects [5–8]. Given the extensive natural diversity of *S*. *marcescens*, it is fitting that this species also has a wide genetic diversity. Multiple studies comparing the genome content of strains from environmental and clinical origins have established the flexible nature of the *S*. *marcescens* genome [9–11], with one study of 45 strains finding that 84% of the total pangenome consisted of unique and accessory genes [12].

The broad genetic repertoire and diverse niche capabilities of *S*. *marcescens* suggest the potential for sublineages with optimized fitness in specific environments or, in the context of human infection, strains with differing pathogenic potential. Indeed, genome sequence analysis indicates that for a large cohort of drug-resistant BSI strains in Europe, infections were primarily caused by a comparatively small number of low-diversity clades, despite a high level of overall genetic distance within the population [11]. Furthermore, differences between clinical and environmental isolates have been described more anecdotally with regard to corneal pathogenesis in a rabbit keratitis model, by the relative lack of prodigiosin pigment production in clinical isolates, the prevalence of certain O and K antigen serotypes, and variations in hemagglutination capability [6,13–15]. Taken together, these observations support the hypothesis that specific lineages of *S*. *marcescens* may be better adapted to causing mammalian infection.

Our previous work determined that fitness of *S*. *marcescens* in an experimental model of BSI was dependent on >200 individual genes in clinical isolate UMH9 [16]. Among these fitness determinants, several capsular polysaccharide (CPS) biosynthesis and translocation genes were identified, and disruption of capsule production resulted in reduced survival of bacteria during infection. Capsule production was also important for resisting the antibacterial activity of human serum. A preliminary genetic comparison of capsule loci (KL) between *S*. *marcescens* clinical isolates originating from BSI indicated substantial sequence heterogeneity at this site [16], consistent with an earlier comparison between two genetically distinct *S*. *marcescens* strains [9]. Though lacking corresponding genetic information, previous biochemical studies have also demonstrated diversity in CPS composition for *S*. *marcescens*, with at least 14 capsular antigens identified by molecular analysis and serotype specificity [17,18]. Given the importance of *S*. *marcescens* CPS in fitness, further investigation into the CPS genetic repertoire of this species is warranted.

The genetic organization of the *S*. *marcescens* KL characterized to date broadly resembles that of *Escherichia coli* Group I capsule and colanic acid loci [19] and the KL of *Klebsiella pneumoniae* [20]. Specifically, the Wza, Wzb, and Wzc proteins that provide polysaccharide export and co-polymerase functions in the Group I and *K*. *pneumoniae* systems (reviewed in [21,22]) have homologs in the annotated KL of *S*. *marcescens* strains UMH9 [16], SM39, and Db11 [9], suggesting a conserved polysaccharide translocation mechanism in the three species. Similarly, the multidrug/oligosaccharidyl-lipid/polysaccharide (MOP) family Wzx lipid flippase (reviewed in [23]) and Wzy polymerase [24] are also expected to be conserved in the *S*. *marcescens* capsule system.

The notion that environmental and clinical isolates of *S*. *marcescens* differ both genetically and phenotypically, together with our observations of CPS involvement in infection, prompted us to determine if there is a relationship between KL type and isolation source. Using a comparative approach, we establish here that there are two predominant KL clades among currently available *S*. *marcescens* clinical isolate genome sequences. The two major infection-associated capsule types that were identified are further differentiated from both environmental capsule clades and other infection isolates by the production of sialic acids.

## Results

### Delineation of surface polysaccharide loci in *S*. *marcescens*

**Fig 1:**
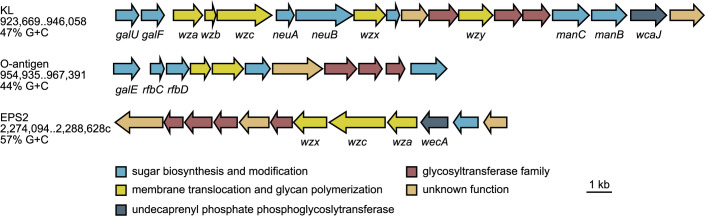
*S*. *marcescens* UMH9 genetic loci with predicted function in surface polysaccharide production. Genes with predicted roles in surface polysaccharide production were identified and manually annotated from the *S*. *marcescens* UMH9 genome sequence. Individual genes (arrows) are colored according to their predicted function. The coordinates and G+C nucleotide content of each locus are indicated.

There are at least three genetic loci in clinical strain UMH9 with the potential to encode variable surface glycans (Fig 1). The lipopolysaccharide (LPS) O-antigen locus and KL are proximal to each other and separated by four genes involved in the production and transport of the S-layer protein SlaA [25], consistent with the arrangement observed in other *S*. *marcescens* isolates [9]. A third locus also appears to encode exopolysaccharide (EPS) synthesis functions and is designated here as EPS2. The EPS2 locus bisects the *nar* respiratory nitrate reductase gene cluster and, similar to KL, contains multiple glycosyltransferase genes as well as homologs of *wza*, *wzc*, and *wzx*. Closer examination of the KL and EPS2 locus reveals differences in the predicted initiating undecaprenyl phosphate phosphoglycosyltransferases associated with each. For the KL, WcaJ is expected to function as an undecaprenyl-phosphate glucose phosphotransferase whereas WecA of EPS2 is an undecaprenyl-phosphate *N*-acetylglucosamine phosphotransferase family member (Fig 1), suggesting that these enzymes transfer different nucleotide sugars to the lipid carrier. Thus, KL and EPS2 are likely to encode for the production of different glycans. Previous work has established that the UMH9 KL is required for the uronic acid-containing surface polysaccharide production [16] that is associated with acidic *S*. *marcescens* CPS [17,18]. The function of the EPS2 locus in UMH9 is presently unknown; however, none of the designated EPS2 genes were identified as significant fitness determinants during murine BSI [16]. Disruption of EPS2 genes was found to reduce *S*. *marcescens* killing of *Caenorhabditis elegans* but failed to demonstrate contributions to virulence for *Drosophila melanogaster*, epithelial cell, or rabbit keratitis infection models [26,27]. It is therefore important to distinguish the EPS2 and KL regions moving forward, given their similarity in gene content but likely independent function. Since the KL has an established role in bacteremia fitness, subsequent analysis was focused exclusively on determining the extent of CPS diversity within the species.

### Capsule genetic diversity within *S*. *marcescens*

**Fig 2:**
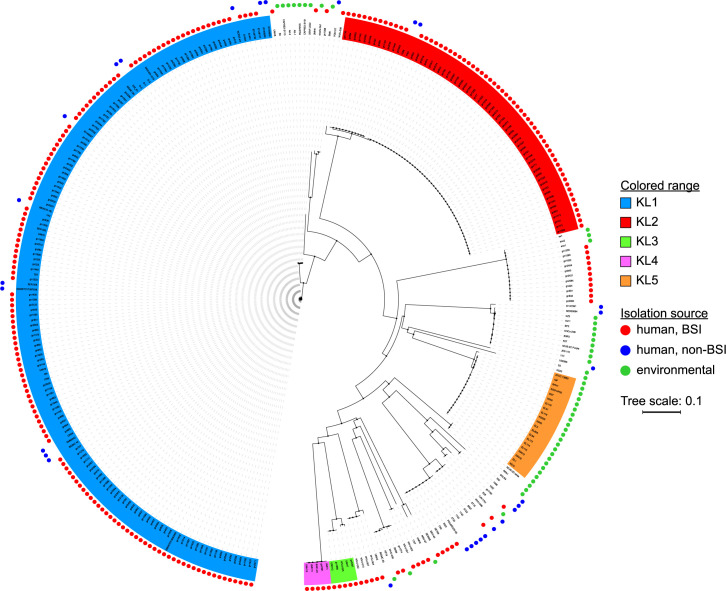
Phylogenetic analysis of the *S*. *marcescens* KL. KL were extracted from *S*. *marcescens* genome sequences and MAFFT alignments were used to generate a neighbor-joining tree. The isolation source was determined from NCBI genome entry metadata and is indicated by colored circles in the outer rings. Selected clades representing different capsule genetic types are shaded and arbitrarily designated KL1-5.

To facilitate a comparison of capsules among multiple *S*. *marcescens* strains, we defined the limits of the KL based on our characterization of the UMH9 strain (Fig 1) and extracted sequences from 324 publicly-available genomes, prioritizing those with high-quality assemblies and available metadata relevant to isolation source (S1 Data). The CPS sequences were aligned and a neighbor-joining tree was generated. Consistent with our initial observations, several clades representing different sequence types were identified (Fig 2). However, there was also a clear over-representation of strains belonging to two clades, designated KL1 and KL2. KL1 and KL2 respectively encompass 152 and 63 isolates and both clades were composed entirely of strains collected from human infections (Fig 2 and S1 Data). This is in stark contrast to KL5 that only consists of environmental isolates, which for the purposes of this study were defined as all isolation sources excluding human infections. With few exceptions, the overall phylogeny supports a strong delineation between infection-associated and environmental KL types within the species. The bulk of the human isolates that were included in the analysis originated from blood culture and there is no discernable pattern to distinguish the BSI isolates from those collected from other infection sites. Two minor infection-associated clades, designated KL3 and KL4, with the greatest genetic distance from KL1 and KL2 were also selected for further analysis.

**Fig 3:**
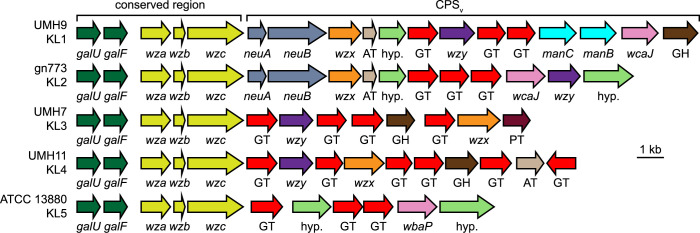
Annotation of KL from selected *S*. *marcescens* strains. KL were identified from the indicated genomes and annotated manually. Individual genes are represented by arrows and were colored according to predicted function. The conserved and variable (CPS_v_) regions of each KL are indicated. Abbreviations: AT, acetyltransferase; GT, glycosyltransferase; GH, glycosylhydrolase; hyp. hypothetical protein of unknown function; PT, pyruvyltransferase.

Individual strains selected from each of the five designated KL types were chosen for a more detailed comparison between loci. Gene-level analysis identified a conserved region present in each strain consisting of predicted UDP-glucose biosynthesis functions (*galU*, *galF*) and polysaccharide polymerization and translocation functions (*wza*, *wzb*, *wzc*) (Fig 3). The adjacent variable region (CPS_v_) exhibited heterogeneous content between each KL type, validating the designations of these strains as having different capsule types. The majority of selected CPS_v_ regions encoded a predicted Wzy polysaccharide repeat polymerase and putative Wzx lipid flippase. The exception to this observation was the ATCC 13880 KL5 strain, which did not appear to encode a Wzx or Wzy homolog. Notably, the genes encoding predicted nucleotide sugar biosynthesis and modification functions also differed substantially between the selected strains.

### Surface attachment of CPS

One intriguing difference between the KL identified here and the characterized capsule genetic regions of other species predicted to share similar CPS assembly and transport pathways, such as *E*. *coli* and *K*. *pneumoniae*, was the absence of a Wzi homolog encoded in either the conserved or variable regions of the *S*. *marcescens* KL. In these systems, Wzi is an outer membrane protein that is required for surface association of CPS and is thought to act in a lectin-like manner by interacting directly with the polysaccharide [28,29]. The presence of *wzi* is also considered to be a differentiating characteristic of Group 1 CPS loci compared to other *E*. *coli* surface glycan loci, such as colanic acid, which often contain genes with high similarity to CPS genes [19]. It was therefore important to test the surface association of acidic CPS in *S*. *marcescens* and identify the gene(s) encoding CPS attachment function. A search for Wzi homologs in strain UMH9 identified a 477 amino acid predicted protein (WP_033636218.1) with up to 63% identity to Wzi proteins of *E*. *coli* and *K*. *pneumoniae*. Intriguingly, the *S*. *marcescens wzi* homolog is encoded at a chromosomal location distal from any of the predicted surface polysaccharide loci (Fig 1). The location of this putative *wzi* homolog (61% G+C) outside of the KL (47% G+C) and the markedly dissimilar nucleotide content of each suggest that these elements have different ancestral origins, with *wzi* having a G+C content that is more similar to the UMH9 genome as a whole (60% G+C).

**Fig 4:**
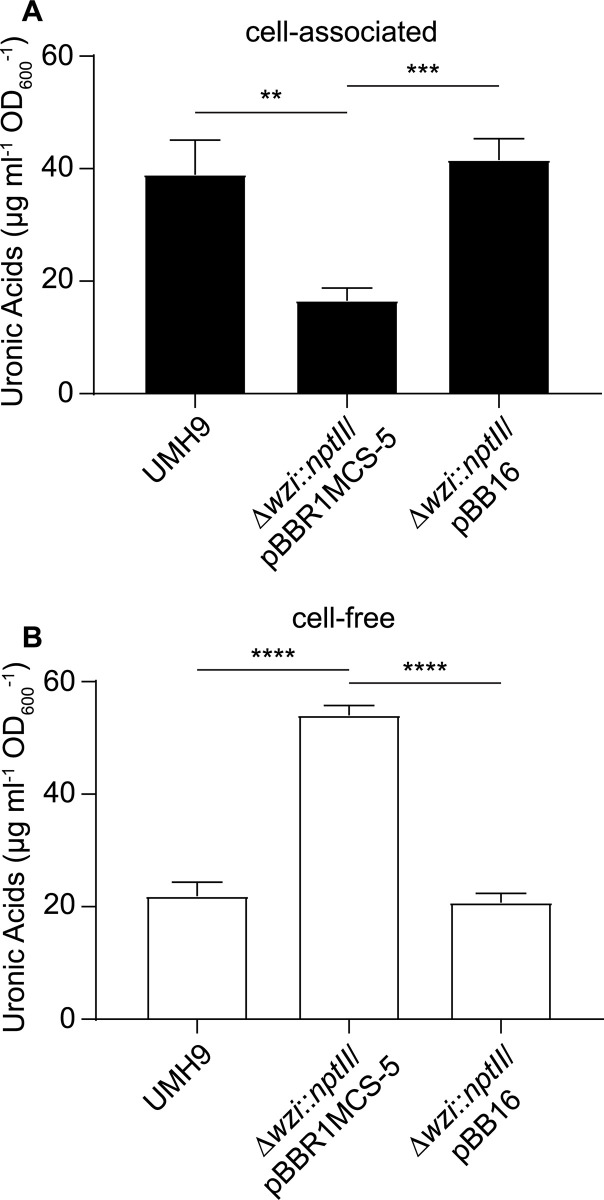
The *wzi* gene is required for cellular association of CPS. Total uronic acids were quantified from *S*. *marcescens* strains cultured in M9 medium supplemented with glucose using a colorimetric assay. Uronic acid levels were normalized to culture density and calculated based on a glucuronic acid standard curve. Cell-associated CPS levels (A) were determined from *S*. *marcescens* strains collected by centrifugation and free CPS levels (B) were determined from conditioned medium cleared of cells by centrifugation and subsequent filtration. An unpaired *t*-test was used to assess significant differences between strains (**, P<0.01; ***, P<0.001; ****, P<0.0001).

**Fig 5:**
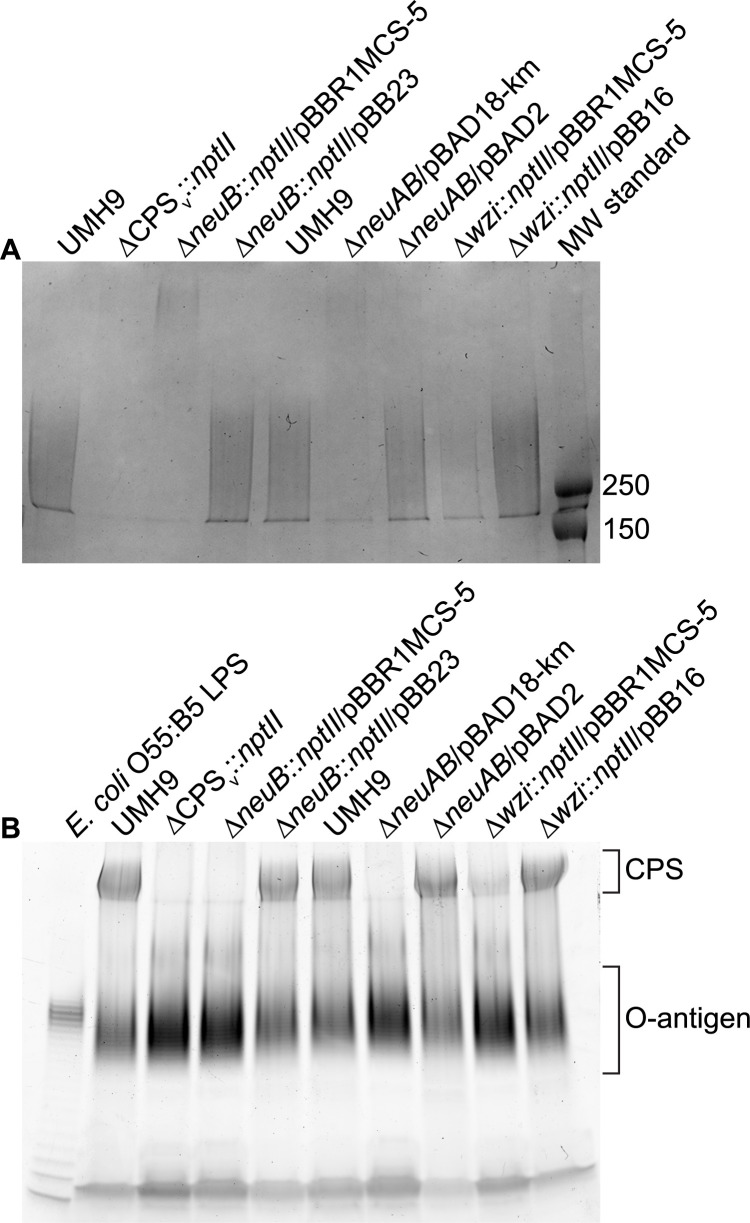
SDS-PAGE of CPS and LPS from *S*. *marcescens* mutant strains. Total cell-associated polysaccharides were isolated from *S*. *marcescens* strains following overnight culture. (A) Purified polysaccharides were resolved on 7.5% SDS-PAGE gels and visualized by Alcian Blue staining. The molecular weights (kDa) of pre-stained protein standards (MW standard) are indicated. (B) Total cell-associated polysaccharides were electrophoresed on 4–20% gradient SDS-PAGE gels. LPS was visualized using the Pro-Q Emerald 300 Liposaccharide Gel Stain Kit with purified LPS from *E*. *coli* serving as a positive control. The region containing the characteristic O-antigen laddering pattern is indicated along with the high molecular weight CPS that co-stains with this procedure.

To determine whether the *S*. *marcescens wzi* homolog contributes to capsule function, *wzi* was disrupted and the levels of cell-associated and cell-free CPS were determined. Cell-associated uronic acid levels that were normalized to culture density were significantly higher (>2-fold, p<0.01) in the wild-type and complemented mutant strains compared to the *wzi* mutant harboring a vector control plasmid (Fig 4A). Likewise, qualitatively lower amounts of high molecular weight polysaccharide were isolated in association with *wzi* mutant cells compared to *wzi*^+^ control strains (Fig 5A). In contrast, a significantly higher amount of uronic acids were detected in cell-free supernatants of the *wzi* mutant compared to the wild-type and complemented mutant strains (Fig 4B). There was no evidence that loss of Wzi function impaired CPS production since the total cell-free plus cell-associated uronic acid levels were approximately equal in strains with or without a functional *wzi* gene. Together, these results establish that cell association of CPS in *S*. *marcescens* is dependent on Wzi, despite the absence of a *wzi* homolog within the KL. The acidic CPS of *S*. *marcescens* should furthermore be considered a *bona fide* surface-bound capsule based on these findings.

### Comparison of exopolysaccharides from selected *S*. *marcescens* strains

**Fig 6:**
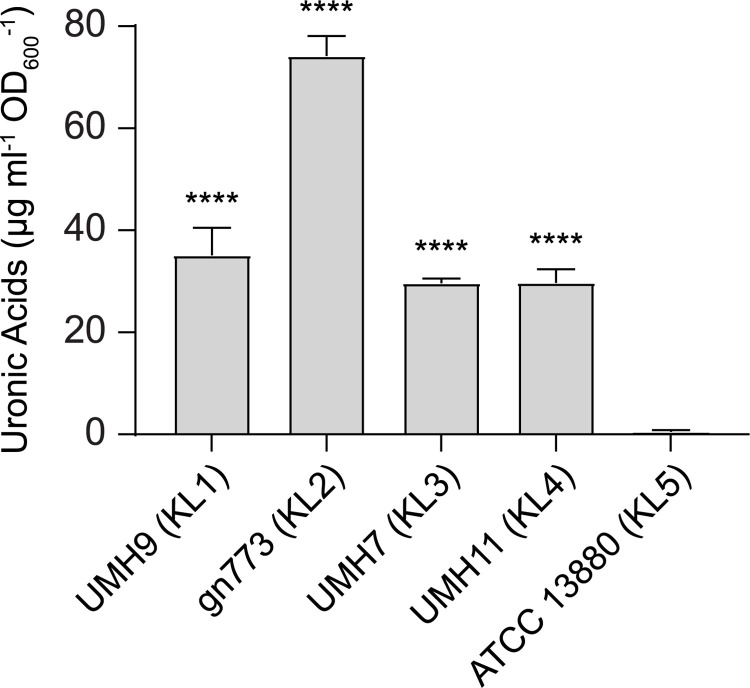
Acidic CPS production by *S*. *marcescens* strains isolated from BSI. Total cell-associated uronic acids from *S*. *marcescens* strains cultured in M9 medium supplemented with glucose were quantified by colorimetric assay. Uronic acid levels were normalized to culture density and calculated based on a glucuronic acid standard curve. Significant differences relative to the acapsular strain ATCC 13880 were assessed by one-way ANOVA with Dunnett’s multiple comparisons test (****, P<0.0001).

**Fig 7:**
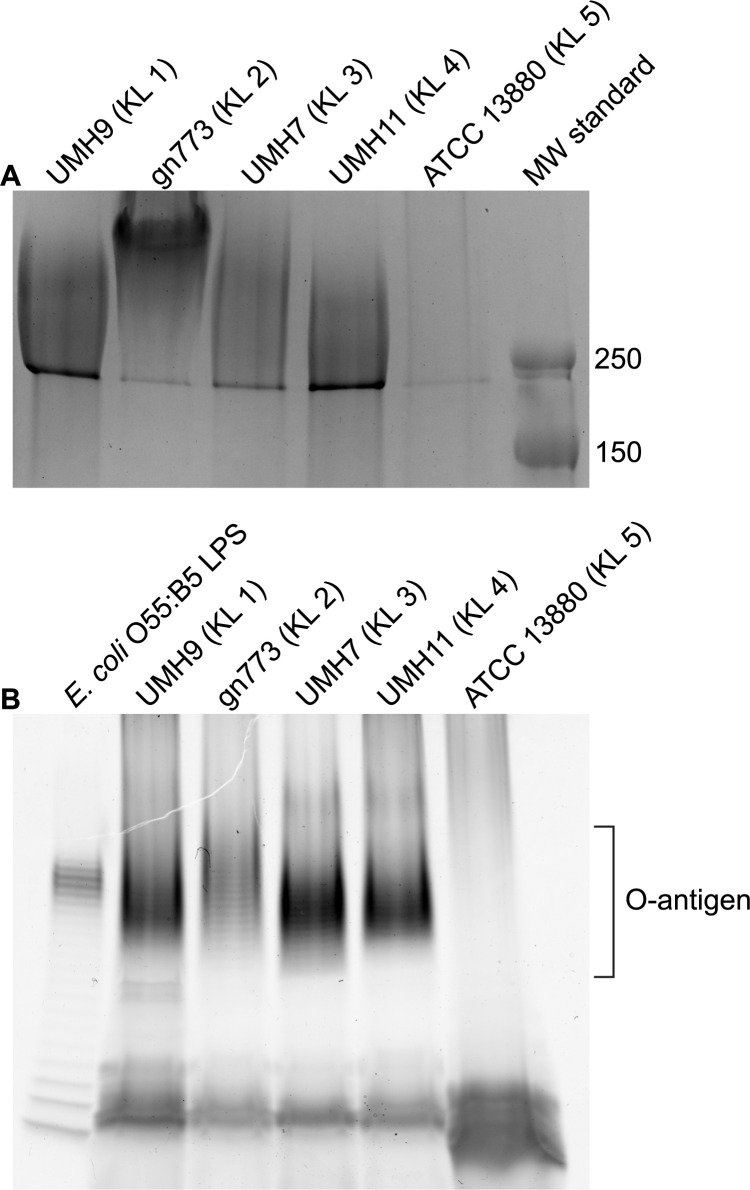
SDS-PAGE of *S*. *marcescens* CPS and LPS from five KL clades. Total cell-associated polysaccharides were isolated from *S*. *marcescens* strains following overnight culture. (A) Purified polysaccharides were resolved on 7.5% SDS-PAGE gels and visualized by Alcian Blue staining. The molecular weights (kDa) of pre-stained protein standards (MW standard) are indicated. (B) Total cell-associated polysaccharides were also electrophoresed on 4–20% gradient SDS-PAGE gels. LPS was visualized using the Pro-Q Emerald 300 Liposaccharide Gel Stain Kit with purified LPS from *E*. *coli* serving as a positive control. The region containing the characteristic O-antigen laddering pattern is indicated.

As an initial assessment of whether each strain representing the five KL types described in Fig 3 were capable of CPS production, isolates were propagated in defined medium and cell-associated uronic acid levels were determined. All tested strains, with the exception of ATCC 13880, yielded uronic acid levels that approximated or exceeded that of the known CPS producer UMH9 (Fig 6). Based on these results, it was concluded that ATCC 13880 does not synthesize CPS under the tested conditions. Conversely, the gn773 KL2 isolate exhibited greater than two-fold higher uronic acid levels compared to UMH9. It was further noted that gn773 has a conspicuously mucoid colony morphology when cultured on solid agar (S1 Fig) that was not observed with any of the other tested strains. This observation, together with the elevated uronic acid levels, suggest a high level of CPS production by gn773. The KL annotations for UMH9, UMH7, UMH11, gn773, and ATCC 13880 (Fig 3) suggest that the CPS produced by these strains are likely different. As an initial test of this prediction, total cell surface polysaccharide was isolated from each strain and resolved by electrophoresis. All strains except again ATCC 13880 yielded abundant high molecular weight polysaccharide when stained with alcian blue, indicative of capsule production (Fig 7A). Interestingly, strain ATCC 13880 also failed to produce the characteristic laddering pattern indicative of O-antigen production when total polysaccharide preparations were stained for LPS (Fig 7B). Therefore, this strain appears to lack the ability to produce either CPS or O-antigen on the cell surface. The subtle differences in migration patterns of Alcian Blue-stained CPS among the tested strains were consistent with the expected variation in capsule composition, though identification of specific composition differences required further investigation.

### Sialic acid synthesis by KL1 and KL2 stains

**Fig 8:**
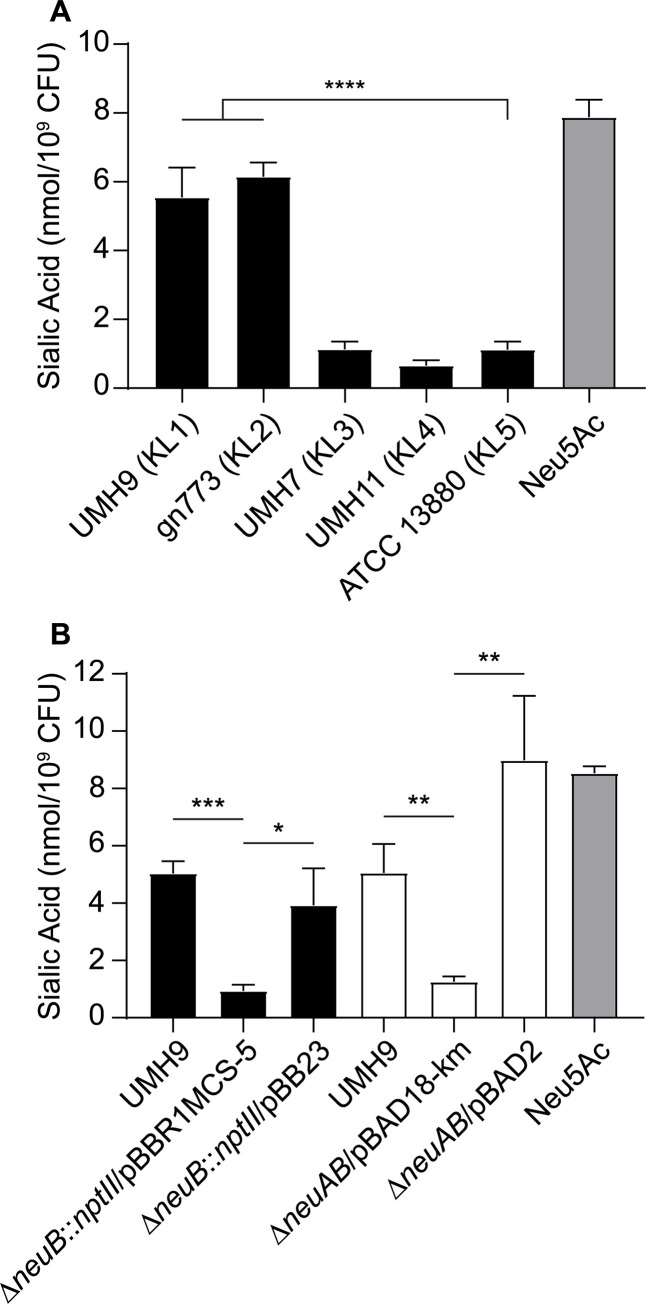
Detection of sialic acids in *S*. *marcescens* strains UMH9 and gn773. Sialic acid production by *S*. *marcescens* strains was measured by thiobarbituric acid assay. Sialic acid levels were normalized to the number of bacteria assayed from each culture and a 100 μM solution of *N*-acetylneuraminic acid (Neu5Ac) was included as a positive control. (A) Differences between strains were assessed by one-way ANOVA with Dunnett’s multiple comparisons test relative to the acapsular strain ATCC 13880 (****, P<0.0001). (B) Statistical significance for the loss of sialic acid production by the Δ*neuB*::*nptII* and Δ*neuAB* mutant strains compared to wild-type and complemented mutants was determined by unpaired *t*-test (*, P<0.05; **, P<0.01; ***, P<0.001).

Strains UMH9 and gn773 each harbor two genes at the start of their respective CPS_v_ regions that were not identified among the other capsule types (Fig 3). These genes, designated here as *neuA* and *neuB*, have predicted protein sequences with similarity to sialic acid biosynthesis proteins of other bacterial species. NeuA is an acylneuraminate cytidylyltransferase family protein (cd02513) with proposed function in formation of CMP-sialic acid sugar-nucleotides that would serve as the substrate for incorporation into the UMH9 and gn773 CPS. NeuB is predicted to be a member of the *N*-acetylneuraminate synthase family (pfam 03102) based on the presence of conserved functional domains. The predicted sequence of NeuB also contains an additional sugar phosphate isomerase/epimerase domain (COG1082) that is not typically observed in other characterized *N*-acetylneuraminic acid (Neu5Ac) synthases, such as NeuB from *E*. *coli* [30,31], suggesting that *S*. *marcescens* NeuB may have additional functional properties. The presence of *neuA* and *neuB* within the UMH9 and gn773 CPS_v_ regions prompted additional efforts to determine whether these strains are capable of sialic acid biosynthesis. A colorimetric biochemical assay was first conducted to determine whether sialic acids could be detected from each strain representing the five KL types. Bacteria cultured in defined medium were subjected to the thiobarbituric acid assay of Warren [32] with Neu5Ac serving as a positive control. UMH9 and gn773 strains were both found to yield significantly higher sialic acid abundance compared to the acapsular ATCC 13880 strain (Fig 8A). UMH7 and UMH11 were also not expected to produce sialic acid based on a lack of *neuA* and *neuB* homologs within their respective KL and only baseline signals from these strains were measured, similar to ATCC 13880.

**Fig 9:**
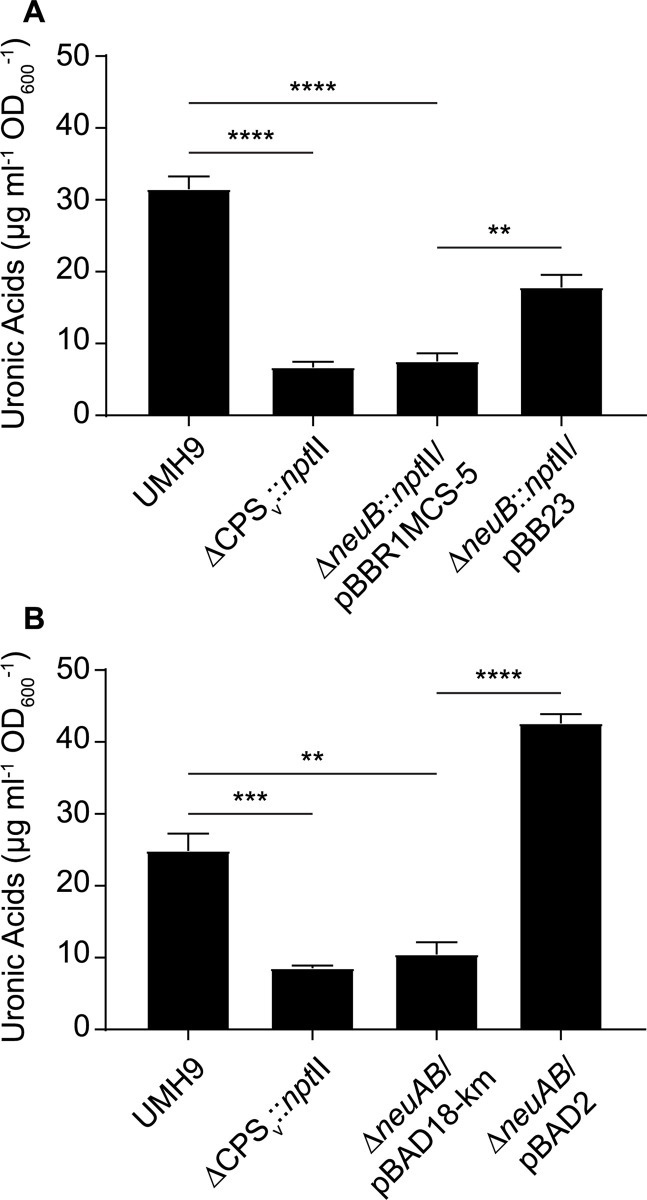
The *neuB* gene is required for CPS synthesis. CPS production by the Δ*neuB*::*nptII* (A) and Δ*neuAB* (B) mutants was evaluated using an uronic acid assay. Cell-associated uronic acids were normalized to the density of the original culture and calculated based on a glucuronic acid standard curve. Statistical significance for differences between strains was assessed by unpaired *t*-test (**, P<0.01; ***, P<0.001; ****, P<0.0001).

To determine whether sialic acid production was dependent on the *neuB* gene identified within the KL1 CPS_v_ region, *neuB* mutant derivatives of UMH9 were generated and tested by thiobarbituric acid assay. Given the high potential for polar effects of *neuB* mutations on downstream CPS_v_ genes, two mutational strategies were pursued. The first mutant harbors an in-frame deletion of the *neuB* open reading frame (ORF) with a kanamycin-resistance gene inserted and the second harbors an unmarked in-frame deletion of both the *neuA* and *neuB* ORFs (Table 1). The absence of either the *neuB* gene alone or both the *neuA* and *neuB* genes resulted in 4-fold lower sialic acid levels in the mutants compared to the parental strain (Fig 8B). In both cases, restoration of sialic acid production was achieved by providing either *neuB* or *neuA* and *neuB* on a multi-copy plasmid, indicating that polar effects of the mutations on downstream CPS_v_ genes were not the cause of sialic acid loss. Based on these results, it was concluded that UMH9 requires the function of *neuB* for sialic acid production. Since disruption of monosaccharide biosynthesis has the potential to disrupt what is expected to be a processive CPS assembly pathway, cell-associated CPS production was also measured for the *neuB* and *neuAB* mutants. In this assay, both sialic acid biosynthesis mutants failed to accumulate high levels of cell-associated uronic acids compared to the UMH9 parent strain or the complemented mutants (Fig 9A and 9B). In fact, the absence of *neuB* resulted in cell-associated uronic acid levels that were not significantly different from a UMH9 mutant derivative lacking the entire CPS_v_ region (ΔCPS_v_::*nptII*). The absence of surface uronic acid association in the *neuB* mutants also correlated with a loss of high molecular weight polysaccharide as determined by alcian blue staining of polysaccharide preparations (Fig 5A). Together, these results demonstrate that sialic acid synthesis is required for CPS production and incorporation onto the cell surface. None of the UMH9 KL mutations tested in this work, including ΔCPS_v_::*nptII*, resulted in loss of O-antigen production in comparison to the UMH9 parent strain when LPS from total polysaccharide preparations was visualized by PAGE (Fig 5B). Thus, O-antigen synthesis is independent of KL gene function.

**Table 1:** *S*. *marcescens* strains and recombinant plasmids used in this study.

Name	Description	Reference
*S*. *marcescens*		
ATCC 13880	pond water isolate	American Type Culture Collection
gn773	human bloodstream infection isolate, also referred to as 2880STDY5682865	[11]
UMH7	human bloodstream infection isolate	[16]
UMH9	human bloodstream infection isolate	[16]
UMH11	human bloodstream infection isolate	[16]
UMH9 Δ*wzi*::*nptII*	1392-bp deletion of the *wzi* ORF and insertion of *nptII* from pKD4	this study
UMH9 ΔCPS_v_::*nptII*	16,214-bp deletion of the capsule variable region and insertion of *nptII* from pKD4	this study
UMH9 Δ*neuB*::*nptII*	Δ*neuB* deletion allele retaining only the *neuB* start and stop codons with *nptII* from pKD4 inserted	this study
UMH9 Δ*neuAB*	in-frame Δ*neuAB* deletion retaining the first 30 bp of *neuA* and the last 30 bp of *neuB*	this study
*plasmids*		
pTOX11_*nptII*_	pTOX11 derivative encoding kanamycin resistance	this study
pBAD2	pBAD18-Km harboring the UMH9 *neuA* and *neuB* ORFs	this study
pBB16	pBBR1MCS-5 harboring the UMH9 *wzi* ORF	this study
pBB23	pBBR1MCS-5 harboring the UMH9 *neuB* ORF	this study

### Polysaccharide composition

**Fig 10:**
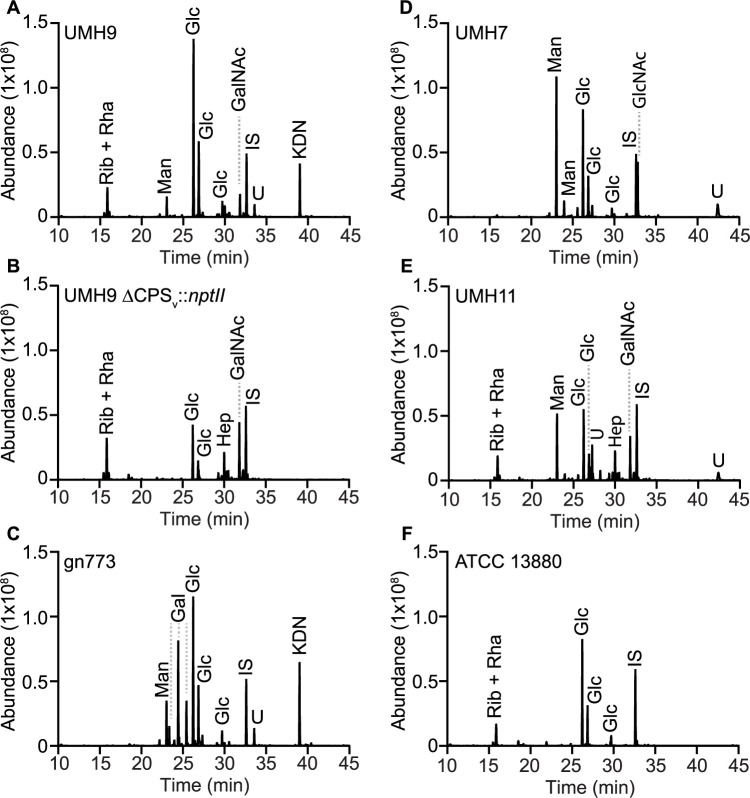
GC-MS of CPS purified from *S*. *marcescens* strains. (A-F) Trimethylsilyl derivatives of CPS monosaccharides were detected by GC-MS. Inositol (1 μg) was included in each run as an internal standard (IS). The ribose and rhamnose peak that appears in some samples is not resolved under the tested conditions. Abbreviations: Gal, galactose; GalNAc, *N*-acetylgalactosamine; Glc, glucose; GlcNAc, *N*-acetylglucosamine; Hep, heptose; KDN, ketodeoxynonulonic acid; Man, mannose; Rha, rhamnose; Rib, ribose; U, unknown.

**Fig 11:**
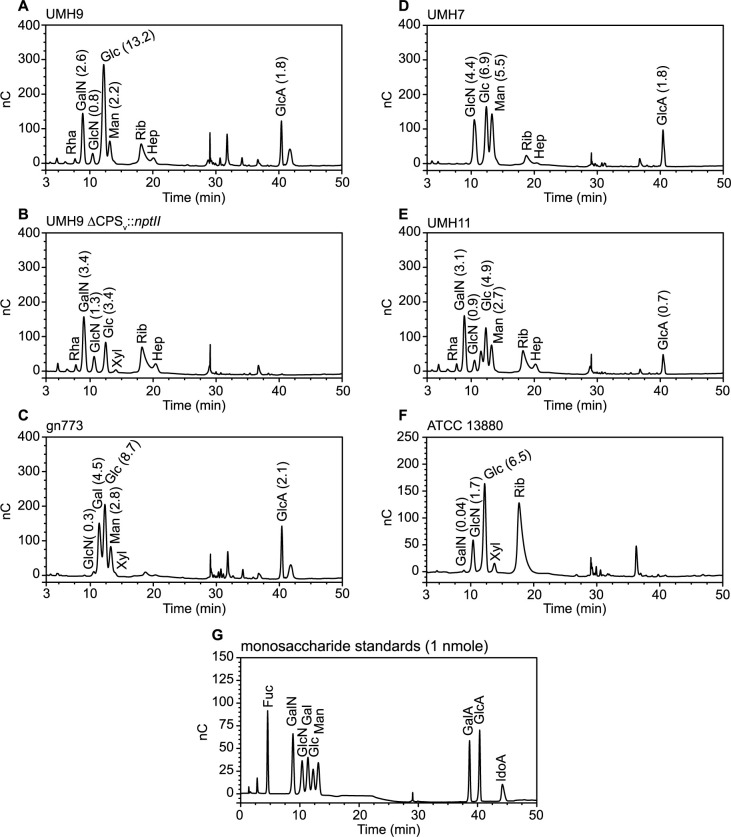
Identification of uronic acids in *S*. *marcescens* polysaccharide preparations. (A-F) Uronic acids and other monosaccharides were detected in 10 μg preparations of *S*. *marcescens* CPS by HPAEC-PAD. The amount (nmole) of monosaccharides detected are shown in parentheses. G. Standards consisting of 1 nmole of selected monosaccharides were assessed simultaneously. Abbreviations: Fuc, fucose; Gal, galactose; GalA, galacturonic acid; GalN, galactosamine; Glc, glucose; GlcA, glucuronic acid; GlcN, glucosamine; Hep, heptose; IdoA, iduronic acid; Man, mannose; Rha, rhamnose; Rib, ribose; Xyl, xylose.

To determine the monosaccharide composition of polysaccharides from each strain, gas chromatography-mass spectrometry (GC-MS) and high-performance anion-exchange chromatography with pulsed amperometric detection (HPAEC-PAD) were conducted on large-scale preparations of soluble polysaccharides. The major peaks identified by the GC-MS method from polysaccharides isolated from the KL1 strain UMH9 corresponded to glucose, mannose, *N-*acetylgalactosamine, and an unresolved ribose + rhamnose signal (Fig 10A). HPAEC-PAD analysis confirmed the identification of these major sugars from UMH9 and additionally demonstrated the presence of glucuronic acid (Fig 11A). Furthermore, GC-MS of UMH9 polysaccharide identified a monosaccharide with a retention time and fragmentation pattern corresponding to the sialic acid 2-keto-3-deoxy-D-glycero-D-galacto-nononic acid or ketodeoxynonulonic acid (KDN), a finding that was subsequently confirmed by an independent HPAEC-PAD run to detect sialic acids using purified KDN as a control (S2 Fig). KDN is a nine-carbon sugar of the sialic acid family that lacks the acetylated amine of the more commonly occurring Neu5Ac and has not been previously observed in *S*. *marcescens*. As a control for the co-purification of non-CPS glycans, composition analysis was also performed on polysaccharides isolated from the UMH9 ΔCPS_v_::*nptII* acapsular mutant. In this strain, mannose and KDN were no longer detected by GC-MS (Fig 10B). The level of glucose detected by HPAEC-PAD in the ΔCPS_v_::*nptII* mutant was also lower (3.4 nmole) than in the parental UMH9 strain (13.2 nmole) when assessed from equivalent of amounts of total polysaccharide (Fig 11A, 11B and S1 Table). Based on these combined analyses, the UMH9 CPS is expected to minimally contain the monosaccharides glucose, mannose, KDN and glucuronic acid. Conversely, the galactosamine, glucosamine, ribose, and rhamnose signals were all present in both the UMH9 and ΔCPS_v_::*nptII* mutant polysaccharides, demonstrating that these components are not dependent on the capsule variable region genes for synthesis and are likely not constituents of the UMH9 CPS. Among the five selected strains, KL2 isolate gn773 has the highest degree of concordance to UMH9 in terms of CPS_v_ genetic organization (Fig 3). In comparison with polysaccharide from UMH9, gn773 also synthesized mannose, glucose, KDN, and glucuronic acid (Figs 10C and 11C and S2). Strain gn773 additionally yielded a galactose signature (Fig 10C) that was quantitated at 4.5 nmole per 10 μg total polysaccharide (Fig 11C). Since galactose was not detected from UMH9, it may serve a key structural difference between the two primary infection-associated capsule types of *S*. *marcescens*. Glucose, mannose, and glucuronic acid were also prominent components of UMH7 (KL3) and UMH11 (KL4) polysaccharides (Figs 10D, 10E, 11D and 11E). Relative to UMH9 and gn773, polysaccharides from both UMH7 and UMH11 yielded a higher mannose:glucose ratio (Fig 11D and 11E) and did not yield detectable levels of KDN (Fig 10D and 10E), consistent with the findings from the colorimetric sialic acid assay. ATCC 13880 (KL5) polysaccharide preparations yielded glucose but neither mannose nor a uronic acid peak (Figs 10F and 11F). The lack of galacturonic acid or glucuronic acid from ATCC 13880 by HPAEC-PAD confirms the similar findings from Fig 6 and solidifies the conclusion that is strain does not synthesize an acidic capsule. Finally, variable levels of glucosamine and/or galactosamine were identified in polysaccharide preparations from all strains, including the two tested acapsular strains (Fig 11A–11F). Assignment of both glucosamine and the galactosamine identified in the UMH9 ΔCPS_v_::*nptII* mutant (Fig 11B) to a specific non-CPS glycan will require further investigation.

**Fig 12:**
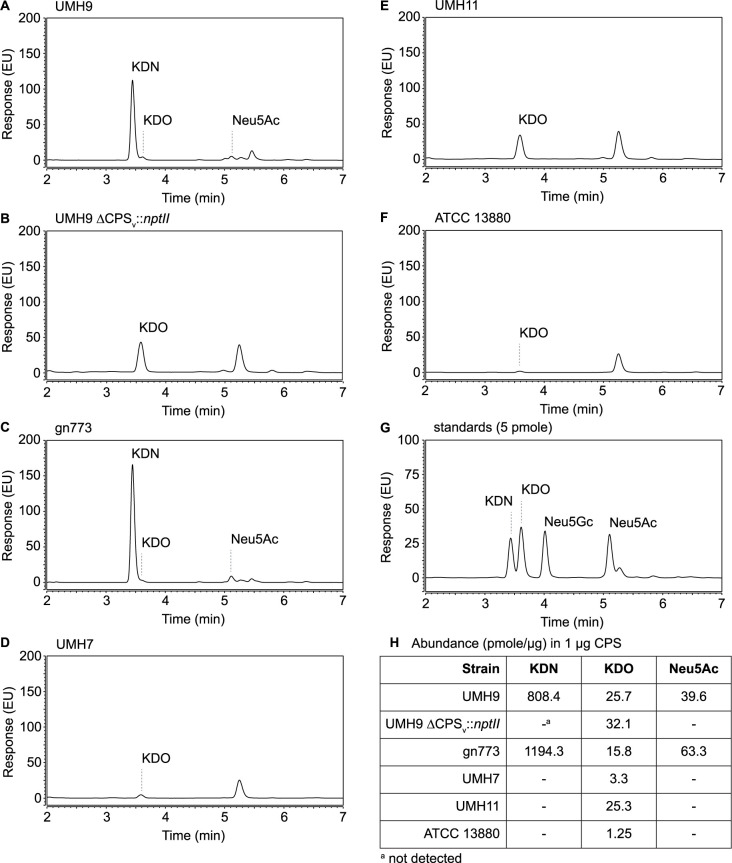
RP-UPLC analysis of sialic acids. (A-F) Polysaccharides were acid hydrolyzed and liberated sialic acids were derivatized with DMB for fluorescence detection following separation by RP-UPLC. (G) RP-UPLC of sialic acid monosaccharide standards (5 pmole). (H) The abundance of selected monosaccharides in each sample was determined based on the standards. Abbreviations: KDN; ketodeoxynonulonic acid; KDO, 3-deoxy-D-manno-octulosonic acid; Neu5Ac, *N*-acetylneuraminic acid; Neu5Gc, *N*-glycolylneuraminic acid.

To further investigate the sialic acid synthesis capability of all selected *S*. *marcescens* strains, hydrolyzed sialic acids were derivatized with 1,2-diamino-4,5-methylenedioxybenzene (DMB) and separated by reversed-phase ultra performance liquid chromatography (RP-UPLC) for fluorescence detection. Specific sialic acids targeted for identification in this assay were KDN, Neu5Ac, and *N*-glycolyneuraminic acid (Neu5Gc) along with the eight-carbon sugar 3-deoxy-D-manno-octulosonic acid (KDO). RP-UPLC of polysaccharides from both gn773 and UMH9 confirmed high levels of KDN in these preparations (Fig 12A and 12C). Consistent with the GC-MS observations, KDN was completely undetectable in the acapsular control strain UMH9 ΔCPS_v_::*nptII* (Fig 12B), as well as the polysaccharides from strains UMH7, UMH11, and ATCC 13880 (Fig 12D–12F). Intriguingly, a low amount of Neu5Ac was also identified from both gn773 and UMH9 polysaccharides (Fig 12A and 12C) at a molar ratio of approximately 1:20 with KDN in both strains (Fig 12H). Neu5Ac was not detected from the UMH9 acapsular control strain (Fig 12B) demonstrating that Neu5Ac, like KDN, is dependent on this genetic region for synthesis. Neu5Gc was not detected in any of the tested strains, consistent with the previously observed lack of Neu5Gc production in bacterial species [33]. All six tested strains yielded low levels (≤32 pmole/μg) of KDO, a common constituent of bacterial LPS that is also found in known *S*. *marcescens* LPS structures [34,35], indicating that LPS was a minor contaminant in polysaccharide preparations.

### Capsule production and fitness

**Fig 13:**
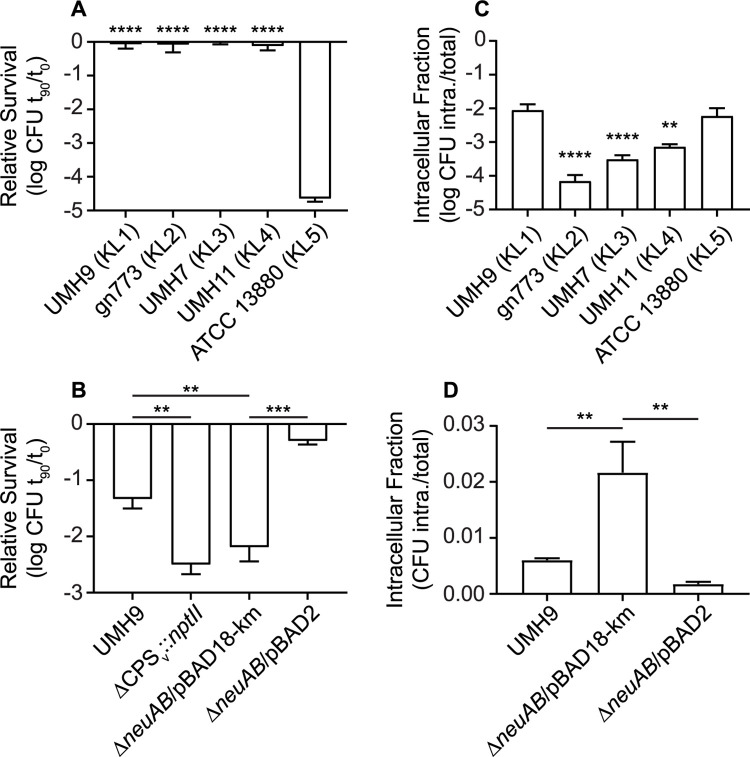
Serum susceptibility and phagocyte internalization of *S*. *marcescens* strains. (A) Bacteria were exposed to 40% pooled human serum for 90 minutes. Viability was calculated relative to the number of bacteria present at the start of the experiment. Differences in serum sensitivity were assessed by one-way ANOVA with Dunnett’s multiple comparisons test relative to strain ATCC 13880. (B) Serum sensitivity of UMH9 capsule mutant strains was determined as described for panel A and an unpaired t test was used to determine statistical significance. (C) Adherent U937 cells were infected with *S*. *marcescens* strains at an MOI of 20. The proportion of intracellular bacteria was determined relative to the total bacterial inoculum after a 1 h treatment with gentamicin to kill extracellular bacteria. A one-way ANOVA with Dunnett’s multiple comparisons test relative to ATCC 13880 was used to assess statistical significance. (D) Internalization of the UMH9 capsule mutant strain by U937 cells was tested as described for panel C. A unpaired *t*-test was used to determine statistical significance. Symbols: **, P<0.01; ***, P<0.001; ****, P<0.0001.

Bacterial capsules represent a critical point of interaction with host defenses and it is hypothesized that *S*. *marcescens* capsule genetic type may contribute to fitness in the infection environment. Each of the five selected strains was exposed to pooled human serum for 90 minutes to test their ability to withstand serum bactericidal activity. All four tested isolates originating from BSI (KL1-4) exhibited minimal loss of viability following serum exposure (Fig 13A). In contrast to the BSI strains, ATCC 13880 was over four orders of magnitude more susceptible to the same serum treatment, which is again consistent with an absence of CPS and O-antigen production. Based on these results, it was concluded that strain ATCC 13880 does not encode compensatory mechanisms of serum resistance in the absence of CPS and O-antigen. As expected, both the UMH9 Δ*neuAB* and ΔCPS_v_::*nptII* mutants were significantly more susceptible to serum compared to the parent strain and the *neuAB*^+^ complemented mutant (Fig 13B), supporting our previously reported findings on the role of capsule in serum resistance [16]. Serum bactericidal activity was also dependent on complement since heat inactivation of serum reduced overall bacterial killing and resulted in no significant differences between wild-type UMH9 and acapsular derivatives (S3 Fig). Further consequences of capsule production were assessed by measuring bacterial internalization when *S*. *marcescens* strains were cultured in the presence of the human monocytic cell line U937. BSI strains gn773, UMH7, and UMH11 all exhibited significantly lower proportions of intracellular bacteria compared to the non-encapsulated ATCC 13880 strain as assessed by gentamicin protection assay (Fig 13C). Interestingly, the UMH9 strain was internalized to a similar degree as ATCC 13880, indicating that the presence or absence of CPS is not the sole determinant of *S*. *marcescens* internalization. However, CPS production does contribute to U937 internalization levels since the UMH9 Δ*neuAB* capsule mutant was >3-fold more sensitive to U937 uptake compared to the wild-type strain (Fig 13D). Since the Δ*neuAB* mutant is deficient in both sialic acid and overall CPS surface display, the specific role for KDN and Neu5Ac in CPS function cannot be determined from these experiments and will require further investigation.

## Discussion

Our analysis of KL diversity among clinical and environmental isolates of *S*. *marcescens* has identified a broad repertoire of genes associated with CPS production within the species. A subset of strains from selected capsule clades were examined further and found to produce distinct polysaccharides, demonstrating the utility of sequence analysis in identifying different capsule types. Despite the overall capacity for *S*. *marcescens* KL diversity, 66% percent of the isolates tested were assigned to just two clades, which were entirely comprised of strains originating from human infection. The infection-associated KL1 and KL2 clades are further distinguished from other capsule types that are encoded by both clinical and environmental isolates by the production of KDN and Neu5Ac. Sialic acid production has not previously been observed for *S*. *marcescens* even though an extensive molecular analysis of O-antigen and CPS glycans from clinical strains was previously conducted, albeit prior to the routine availability of *S*. *marcescens* genome sequences [18]. Whether the KDN and Neu5Ac production by KL1 and KL2 strains reported here represents a recent emergence of novel capsule types or whether KDN-producing strains were simply not included in the prior serotype analysis remains to be determined. Finally, our results establish that *S*. *marcescens* strains differentiated by their capsule genetic type have variations in their ability to withstand the bactericidal activity of human serum and internalization by monocytic cells, indicating the potential for capsule type to influence bacterial fitness.

Horizontal transfer of bacterial capsule biosynthesis genes has frequently been speculated as one of the primary mechanisms for generating inter- and intra-species diversity within CPS-encoding regions. The *S*. *marcescens* CPS_v_ region is flanked by two comparatively well-conserved loci, the capsule genes spanning from *galU*-*wzc* (Fig 3) and the four-gene S-layer locus. This arrangement could facilitate a role for recombination in the generation of capsule locus diversity and may allow for diversification of capsular genes at a rate that is independent of other genomic loci [36,37]. The *S*. *marcescens* Wzi homolog identified here contributes to the cell-association of CPS in a manner that is consistent with the proposed function of Wzi in assembling or tethering translocated CPS at the bacterial surface of *E*. *coli*, *K*. *pneumoniae*, and *Acinetobacter baumannii* [28,29,38]. In each case, when *wzi* is disrupted CPS no longer tightly associates with the cell surface and is found secreted in the supernatant. Interestingly, the localization of *wzi* outside of the *S*. *marcescens* KL and the striking difference in nucleotide content between *wzi* and the UMH9 KL strongly suggest different evolutionary origins for these functionally-related loci. This arrangement furthermore suggests that these loci are likely to be independently variable. More specifically, *S*. *marcescens wzi* would not be subject to putative homologous recombination events that would facilitate exchange of KL between organisms. This is an intriguing notion considering that *E*. *coli* Wzi directly interacts with capsule polymers [29]. How then is *S*. *marcescens* capsule variation accommodated at the level of surface association? One possibility is that *S*. *marcescens* CPS, while clearly variable based on the findings presented here, may have a sufficiently conserved structure between types to facilitate a non-KL-specific Wzi interaction. Alternatively, a currently unrecognized mechanism for Wzi variation may facilitate CPS type-specific interactions or the degree to which cell association of CPS occurs may vary between capsule type.

A large number of the genomic sequences used for our KL phylogenetic comparison were characterized as part of a 10-year study of drug-resistant BSI *S*. *marcescens* isolates collected by 31 hospitals within the United Kingdom and Ireland [11]. One conclusion from this study was that genetically similar isolates, which the authors separated into nine major clades, had emerged from the broader population. While the inclusion of these MDR BSI isolates in our current study may impact the total diversity of capsule types observed, it is important to note that the two predominant CPS clades were not composed exclusively of strains from this cohort. In fact, the largest capsule clade KL1 harbored isolates from nine countries on four continents and included multiple strains from our own University of Michigan BSI collection (S1 Data). Moreover, both KL1 and KL2 clades included strains that were isolated from non-BSI infections. Together, these observations support our conclusion that KL1 and KL2 represent significant infection-associated capsule types but does not exclude the possibility that additional infection-associated capsule types may be identified as more broadly representative sequence data become available. For instance, clades KL3 and KL4 were also exclusive to *S*. *marcescens* originating from BSI but the currently low number of representative strains (n = 5) in each clade limits a broader interpretation. The environmental *S*. *marcescens* strains included in the present study originated from widely different sources (S1 Data), yet there was also a distinct clustering of these stains within our KL phylogeny (Fig 2). One interpretation for the independent clustering of clinical and environmental isolates is that specific capsule types may provide a fitness advantage in either the infection environment or the natural environment. CPS production may alternatively be subject to negative selection among environmental isolates, ultimately resulting a higher frequency of acapsular strains. Further assessment of additional environmental strains will be needed to distinguish these possibilities and determine whether environmental isolates are predominantly acapsular, as was observed for the ATCC 13880 KL5 strain tested here. It is also possible that the considerable range of habitable environments by *S*. *marcescens* may prevent a true delineation between clinical and environmental strains and that more inclusive genetic correlations are needed to fully inform the fitness potential of a *S*. *marcescens* strain in a given environment.

The annotation of KL1 and KL2 (Fig 3) led us to hypothesize that strains belonging to these clades encoded the ability to produce a sialic acid since NeuA and NeuB have homology to other bacterial sialic acid-related capsule enzymes. This prediction was validated first by biochemical assay, demonstrating that strains UMH9 and gn773 produced an undefined sialic acid at levels not detected in the other tested strains (Fig 8). Further molecular analysis of polysaccharides from UMH9 and gn773 subsequently identified KDN and Neu5Ac as the sources of the sialic acid signal in these strains (Figs 10 and 11). It remains to be determined what proportion of the overall *S*. *marcescens* population produces KDN and Neu5Ac, but the phylogenetic analysis suggest that this trait could be widespread among clinical isolates belonging to clades KL1 and KL2. Neither KDN nor Neu5Ac were detected from control polysaccharide preparations of the UMH9 ΔCPS_v_::*nptII* acapsular mutant (Figs 10B and 11B) and thiobarbituric acid assay also confirmed that loss of sialic acid production resulted from disruption of *neuB* (Fig 8). Thus, synthesis of both KDN and Neu5Ac are dependent on KL genes. Although bacterial and eukaryotic sialic acid synthesis are accomplished via distinct pathways, it is interesting to note that the human Neu5Ac synthase is capable of producing phosphorylated forms of both Neu5Ac and KDN [39]. The observation that KDN and Neu5Ac were recovered at an approximately 20:1 ratio from polysaccharide preparations of strains UMH9 and gn773 is intriguing and it may be unlikely that KDN and Neu5Ac occur at this ratio within a single repeating unit of the CPS. One possible explanation for the observed arrangement is that KDN and Neu5Ac could be constituents of different exopolysaccharides. While we do not exclude this possibility, the fact that synthesis of both sialic acids are dependent on KL genes suggests that both monosaccharides are associated with CPS function. An alternative explanation is that Neu5Ac may be substituted for KDN in the CPS at low frequency under the conditions tested. The similar structures of KDN and Neu5Ac support this hypothesis as do data from other investigators who have demonstrated the ability to manipulate incorporation of different sialic acids into a single bacterial glycan based on monosaccharide availability [40–42].

The first published report of KDN in a bacterial CPS was from Knirel *et al*. who observed that KDN was produced by a *Klebsiella ozaenae* serotype K4 strain [43]. KDN has since been identified in the CPS of a thiamine auxotroph of *Sinorhizobium fredii* [44] and *in vitro* KDN synthesis was reported using purified proteins originating from *Bacteroides thetaiotaomicron* [45]. In the latter case, KDN was speculated to be incorporated into one of the multiple CPS encoded by this species [45,46]. Bacterial KDN incorporation into cell wall glycans has also been reported for multiple *Streptomyces* species [47,48]. Despite these examples, KDN-containing CPS appear to be uncommon among bacteria, and with the possible exception of *K*. *ozaenae* and now *S*. *marcescens*, KDN production by human pathogens has been sparsely reported. What then is the role of this understudied sialic acid in CPS function? KDN is produced by the human host [39,49] but the biological function of KDN in this context is not fully understood. Conversely, Neu5Ac is an abundant constituent of host cell surface glycans [50] and has also long been recognized as an important component of certain bacterial CPS glycans. Well-studied examples include the demonstration of Neu5Ac as a terminal residue of group B *Streptococcus* CPS (reviewed in [51]) or as the sole constituent in the polysialic acid structures of K1 *E*. *coli* and serogroup B *Neisseria meningitidis* [52,53]. Given that Neu5Ac, and possibly KDN, are produced by host cells and may be recognized as a self-antigens in the context of bacteria, there is potential for these sialic acids to serve in “camouflaging” the *S*. *marcescens* surface and reducing efficiency of recognition by the immune system. This molecular mimicry [54] by bacterial surface polysaccharides, specifically those containing Neu5Ac, has been shown to modulate the immune response through engagement of Siglec receptors in the case of sialylated *Streptococcus* CPS [55,56]. Whether or not *S*. *marcescens* CPS can function in an immuno-modulatory capacity remains to be determined. Interestingly, a recent report indicates that KDN-containing bacterial glycans may not be as immunologically protected as those containing Neu5Ac [42].

In conclusion, we have demonstrated that a large number of sequenced *S*. *marcescens* strains isolated from human infection can be classified into two clades based on capsule genotype. *S*. *marcescens* infection isolates and environmental isolates were also largely segregated based on KL genotype alone. Finally, the identification of sialic acids KDN and Neu5Ac among *S*. *marcescens* BSI isolates represents a novel finding that may impact our understanding of the interactions between *S*. *marcescens* and the human host.

## Materials and methods

### Bacterial strains and culture conditions

The *S*. *marcescens* strains used in this study are described in Table 1. *E*. *coli* DH10B and Top10 were used for routine cloning purposes. *E*. *coli* strain S17-1 λ *pir* [57] was used as a donor strain in conjugations. *S*. *marcescens* strains were routinely cultured in LB medium [58] or M9 medium [59] supplemented with 1 mM MgSO_4_, 36 μM FeSO_4_, 100 μM CaCl_2_ and 20 mM glucose, as indicated. Antibiotics for bacterial culture were used at the following concentrations: kanamycin, 50 μg/mL; gentamicin, 10 and 20 μg/mL; spectinomycin, 100 μg/mL.

### Generation of *S*. *marcescens* mutant strains

All oligonucleotide primers used in the construction of mutant alleles are listed in S2 Table. For construction of the Δ*neuB*::*nptII* mutant, a UMH9 *neuB*-containing segment that included 128-bp of upstream and 133-bp of downstream sequence was PCR amplified (oligonucleotides SDH_P1 and SDH_P2) with Easy A polymerase (Agilent) from UMH9 genomic DNA and TOPO-cloned into pCR2.1-TOPO (Invitrogen). Oligonucleotides SDH_P3 and SDH_P4 were used to amplify the recombinant plasmid and generate a *neuB* in-frame deletion into which the *nptII* kanamycin resistance gene, amplified (SDH-P5 and SDH_P6) from pKD4 [60], was inserted by NEBuilder HiFi DNA Assembly reaction (NEB). Recombineering-based mutagenesis of UMH9 harboring pSIM19 [61] was performed as previously described [16,62] with a 1056-bp Δ*neuB*::*nptII* linear amplicon, which was PCR amplified (oligonucleotides SDH_P1 and SDH_P2) from pCR2.1-Δ*neuB*::*nptII*. The UMH9 ΔCPS_v_::*nptII* mutant strain was also generated by recombineering. Oligonucleotides with 5′ sequence homology to the 50-bp immediately upstream of the *neuA* start codon (MTA_P1) and the last 50-bp of the BVG96_RS04590 open reading frame (MTA_P2) were used for amplification of the *nptII* gene from plasmid pKD4. The PCR product was digested with DpnI to remove plasmid template and transformed into UMH9/pSIM19 by electroporation. The recombinant clone was screened by PCR for the presence of a 16,214-bp deletion in the CPS_v_ region and insertion of the *nptII* gene. For generation of the UMH9 *wzi*::*nptII* mutation, oligonucleotides (MTA_P3 and MTA-P4) with 5′ sequence homology to the *wzi* open reading frame were used to amplify the *nptII* kanamycin resistance gene from pKD4. The resulting product was DpnI-digested to remove template DNA and used to transform strain UMH9/pSIM19 by electroporation. Recombinant clones having a 1392-bp deletion of *wzi* and insertion of the *nptII* gene were identified by PCR. All mutant strains generated by recombineering were cured of plasmid pSIM19 prior to phenotypic analysis.

The unmarked Δ*neuAB* mutation was generated by allelic exchange using a derivative of suicide plasmid pTOX11 [63] that harbored the *nptII* resistance gene in place of *aacC1*. Plasmid pTOX11 was amplified by PCR using oligonucleotides MTA_P5 and MTA_P6 to generate a linear fragment lacking the *aacC1* gene. The *nptII* fragment from plasmid pKD4 was PCR-amplified with oligonucleotides MTA_P7 and MTA_P8 having 5′ homology to pTOX11. The two fragments were joined by HiFi DNA Assembly to create pTOX11_*nptII*_. To generate the Δ*neuAB* allele in pTOX11_*nptII*_, pTOX11_*nptII*_ was amplified with oligonucleotides MTA_P9 and MTA_P10 and digested with DpnI and XmaI (NEB) then combined by HiFi Assembly with a 750-bp amplicon containing 3′ end of *wzc* and the first 30 bp of the *neuA* ORF (oligonucleotides SDH_P7 and SDH_P8) and a second 750-bp amplicon containing the last 30 bp of *neuB* open reading frame and 5′ end of *wzx* (oligonucleotides SDH_P9 and SDH_P10). The resulting pTOX11_*nptII*_ + Δ*neuAB* plasmid was confirmed by sequencing and transformed into *E*. *coli* S17-1 *λpir* for conjugation with recipient strain UMH9. Allelic exchange was performed as described previously [63]. UMH9 transconjugants harboring the Δ*neuAB* allele were identified by PCR. The absence of Mu phage, originating from the S17-1 donor strain, in the UMH9 Δ*neuAB* mutant was assessed using the PCR screening strategy of Ferrieres *et al*. [64]. All *S*. *marcescens* strains described in this work were verified by sequence analysis.

### Genetic complementation

All oligonucleotide primers used in the construction of complementation plasmids are listed in S2 Table. To construct the *neuB* complementation vector pBB23, the 2241-bp open reading frame of *neuB* including 9-bp of upstream non-coding sequence was amplified from UMH9 genomic DNA using oligonucleotides SDH_P11 and SDH_P12. The pBBR1MCS-5 plasmid was also amplified with oligonucleotides SDH_P13 and SDH_P14, then treated with DpnI- and ClaI to digest template DNA. The two fragments were joined by HiFi DNA Assembly and transformed into electrocompetent *E*. *coli* DH10B cells. For construction of *neuAB* complementation vector pBAD2, the *neuA* and *neuB* genes, including 119-bp upstream non-coding sequence, were PCR amplified from UMH9 genomic DNA template using oligonucleotides SDH_P15 and SDH_P16. Plasmid pBAD18-kan was amplified with oligonucleotides SDH_P17 and SDH_P18, then DpnI- and XbaI-treated to remove template DNA. The two linear fragments were joined by HiFi Assembly and electroporated into *E*. *coli* DH10B. Complementation of the Δ*wzi*::*nptII* mutation was accomplished by first amplifying a 1659-bp fragment containing the *wzi* open reading frame with oligonucleotides MTA_P11 and MTA_P12 followed by digestion of the product with KpnI and HindIII restriction enzymes. The digested fragment was ligated to pBBR1MCS-5 linearized with the same restriction enzymes and transformed into competent TOP10 cells. All recombinant plasmids were confirmed by sequencing and introduced into the respective UMH9 recipient strains by electroporation.

### Capsule locus phylogenetics

All KL sequences were extracted from publicly available *S*. *marcescens* genomes. Isolation source metadata linked to NCBI genome entries was used to categorize *S*. *marcescens* strains as originating from BSI, non-BSI human infections, or environmental sources. KL were initially located by BLAST using the Wza sequence of strain UMH9 (WP_033637739.1). Putative loci were further refined based on genomic context due to the presence of multiple Wza homologs in many *S*. *marcescens* genomes. For the purposes of this study, the *galU* gene encoding a predicted glucose-1-phosphate uridylyltransferase enzyme was designated as the first gene in each KL. The terminus of each KL was defined as the last *galU*-proximal ORF immediately adjacent to the conserved S-layer protein gene *slaA* [25], regardless of orientation. Accession numbers and sequence coordinates for all CPS-encoding regions identified in this study are available in S1 Data. KL identified by the described method, but spanning multiple contigs, were excluded from the analysis as were KL from genomes containing >300 contigs. Identified KL were aligned with MAFFT v. 7 [65] using the FFT-NS-i strategy. A neighbor-joining tree was constructed using gap-free sites and Jukes-Cantor substitution model. Bootstrap resampling was set to 100. The resulting tree was annotated using the iTOL annotation editor [66].

### Uronic acids quantification

Extracellular uronic acids were quantified from 4 mL overnight cultures of *S*. *marcescens* as previously described [16,67–69]. Cell-associated uronic acids were measured from bacteria collected by centrifugation. Briefly, bacterial pellets were resuspended in PBS, combined with Zwittergent 3–14 (1%), and incubated at 50°C for 20 min. Following centrifugation, two identical aliquots of supernatant were transferred to microcentrifuge tubes to serve as experimental and no-reagent control samples. Total polysaccharides were precipitated by the addition of ethanol (80%) and recovered by centrifugation. The resulting precipitate was dissolved in water. Sodium tetraborate (0.0125 M) in concentrated sulfuric acid was added to each sample and incubated at 100°C for 5 minutes. After cooling, the experimental samples received freshly prepared 3-hydroxydipenyl solution (0.15% 3-hydroxydiphenyl, 0.5% sodium hydroxide) while an equal volume of vehicle was added to no-reagent control samples. The optical density (520 nm) of each sample was determined and non-specific reactivity was corrected for by subtracting the OD_520_ values of the no-reagent controls. Uronic acids levels were determined from a standard curve consisting of glucuronic acid. All values were normalized to the starting optical density (600 nm) of each culture.

Where indicated, cell-free uronic acids were measured in parallel with the cell-associated uronic acids. Spent culture supernatants were collected and subjected to a second centrifugation, followed by filtration (0.22 μm, Millipore) to completely remove bacterial cells. Filtered supernatants were mixed with ethanol (80%) and incubated at 4°C overnight. Following centrifugation of precipitated polysaccharides, the supernatant was removed completely and the pellets were rehydrated in water. The samples were then processed as described for cell-associated uronic acid quantification.

### Serum resistance

Resistance of *S*. *marcescens* strains to the bactericidal activity of human serum was determined using pooled complement serum (Innovative Research). Bacterial strains were cultured overnight in M9 medium then washed 1x in PBS prior to assay. A bacterial suspension containing 2 x 10^6^ CFU was exposed to a 40% dilution of serum in PBS in a total volume of 0.2 ml for 90 minutes at 37°C. Control experiments to test for the requirement of complement in bactericidal activity were conducted with serum that had been heat-inactivated at 56°C for 45 min. The results are reported as the number of viable bacteria present at 90 minutes relative to the number present at the start of the experiment and are the mean from three biological replicates.

### Sialic acid assay

Sialic acid produced by *S*. *marcescens* was measured using a modified thiobarbituric assay from Warren [32]. Bacteria (0.5 ml) from overnight cultures were washed with PBS then resuspended in 0.1 ml of PBS. The suspension was subjected to acid hydrolysis with 0.1 N HCl at 80°C for 60 min. Following centrifugation, the supernatant was treated with 10% trichloroacetic acid, vortexed, and incubated on ice for 10 min. Precipitate was removed by centrifugation and the supernatant was treated with 0.2 M sodium periodate in 9.0 M phosphoric acid for 20 min. A solution containing 10% sodium arsenite, 0.5 M sodium sulfate, and 0.1 N H_2_SO_4_ was added. Samples were vortexed until the disappearance of any yellow-brown color and incubated at room temperature for 2 min. A thiobarbituric acid solution (0.6% thiobarbituric acid and 0.5 M sodium sulfate) was added and incubated at 100°C for 15 min. Once cooled, the resulting chromophore was extracted into an equal volume of cyclohexanone and allowed to stand for 5 min at room temperature for phase separation. Absorbance of the organic phase was measured in quartz cuvettes at a wavelength of 549 nm. The amount of sialic acid was determined with the following formula: (Volume (mL) prior to extraction x OD_549_) / 57 = μmoles Neu5Ac equivalents. The amount of sialic acid detected was normalized to the number of bacteria (CFU/ml) assayed and 100 μM Neu5Ac served as the positive control. Results are reported as the mean from three biological replicates.

### Polysaccharide purification and PAGE

Routine extraction of total polysaccharide from *S*. *marcescens* was based on published methods [70]. Overnight cultures were normalized by OD_600_, followed by collection of bacteria by centrifugation. The pellet was resuspended in 200 μL of lysis buffer (60 mM Tris-HCl pH 8.0, 50 μM CaCl_2_, and 10 mM MgCl_2_) containing 3 mg/mL lysozyme, incubated at 37°C for 1 hr, and vortexed thoroughly. Bacterial suspensions were frozen (-80°C) and thawed (37°C) three times. The samples were treated with DNase I and RNase A for 1 hr at 37°C. Sodium dodecyl sulfate was added and incubation was continued for 30 min. Lysates were boiled for 10 min and cooled to room temperature. Lysis buffer containing proteinase K was then added to the solution and incubated at 60°C for 1 hr. The lysate was cleared by centrifugation at 13,000 rpm for 2 min and supernatant was transferred to a new tube. Polysaccharides were precipitated overnight at -20°C following addition of ethanol. Polysaccharide precipitate was recovered by centrifugation and the ethanol was removed completely before proceeding. Recovered polysaccharide was rehydrated in 35 μl of SDS-PAGE loading buffer and boiled for 10 min at 100°C. For visualization of CPS, 15 μl of sample was loaded on a 7.5% precast SDS-PAGE gel (Bio-Rad) and electrophoresed at 150V. Gels were rinsed 6 times in distilled water for 10 min before staining with Alcian Blue solution (40% ethanol, 55% dH_2_O, 5% glacial acetic acid, 0.1% Alcian Blue). De-staining was carried out in the same solution lacking Alcian Blue. Gels were rehydrated in water prior to imaging. LPS visualization from the same preparations was carried out on a 4–20% precast SDS-PAGE gels (Bio-Rad) electrophoresed at 150V. Gels were stained using the Pro-Q Emerald 300 Liposaccharide Gel Stain Kit (Molecular Probes).

### Monosaccharide analysis

Large-scale polysaccharide preparations were obtained from 1 L of overnight growth from M9 glucose cultures. Bacteria were collected by centrifugation, resuspended in 50 ml of distilled water, and added to an equal volume of pre-warmed 90% phenol. The extraction was performed at 65°C for one hour, then the mixture was allowed to stand overnight at 4°C. The aqueous phase was collected following centrifugation and dialyzed extensively against distilled water using a 10,000 MW slide-a-lyzer (ThermoFisher). Dialyzed samples were lyophilized to dryness followed by rehydration in TRIS buffer and successive treatment with RNaseA, DNase I, and proteinase K. A second extraction in 90% phenol was performed followed by extensive dialysis of the aqueous phase. Insoluble material was removed by centrifugation at 150,000 x *g* for 2 hours. The CPS-containing supernatant was recovered and lyophilized to dryness for subsequent analysis.

GC-MS, RP-UPLC, and HPAEC-PAD analyses were performed by the University of San Diego GlycoAnalytics Core facility. For GC-MS, 25 μg of each CPS was methanolized at 80°C for 16 h followed by re-*N*-acetylation and derivatization with trimethylsilyl. GC-MS was performed with EI-mode using a Restek-5ms capillary column. For sialic acid detection, CPS samples were dissolved in 2 M acetic acid and heated to 80°C for 3 hours to release sialic acids. Released sialic acids were collected by ultra-filtration through a 3 kDa molecular weight cut off filter. For HPAEC-PAD, the standard consisted of 1 nmole of purified KDN. Hydrolyzed sialic acids from polysaccharide preparations were also derivatized with DMB and analyzed by RP-UPLC against 5 pmole of purified KDO, KDN, Neu5Ac, and Neu5Gc as standards. A uronic acid analysis was also performed on each CPS preparation. 10 μg of each sample was treated with 2 M trifluoroacetic acid at 100°C for six h followed by acid removal. Samples were co-evaporated with isopropanol twice and dissolved in water for analysis by HPAEC-PAD. Selected monosaccharides (1 nmole) were included as standards.

### Phagocytosis assays

Prior to the start of the experiment, non-adherent human U937 cells (ATCC) were cultured for 2–3 days under 5.0% CO_2_ at 37°C in RPMI medium (Corning) supplemented with 10% Fetal Bovine Serum (FBS) (Invitrogen). Cells were counted and distributed to a 75 cm^2^ Nunc Cell Culture Treated EasYFlask (Thermo Scientific) and phorbol myristate acetate (Sigma) was added to a final concentration of 0.2 μg/ml. U937 cell activation was carried out for 48 h under these conditions. Adherent U937 cells were released from the flasks and seeded at a final density of 1 x 10^6^ cells/mL in RPMI + 10% FBS into 24-well cell culture plates (Corning Costar), which were incubated at 37°C for 2 hr. Washed bacteria from overnight cultures in M9 medium were resuspended in RPMI + 10% FBS and used to inoculate U937 cells at a MOI of 20. Cells inoculated with bacteria were centrifuged for five minutes at 200 x *g*, then incubated at 37°C for 30 minutes. Non-adherent bacteria were removed by aspiration and wells were washed 3x with DPBS. RPMI + 10% FBS containing 100 μg/ml gentamicin was added to the wells and allowed to incubate for one hour to kill any extracellular bacteria. The medium was aspirated and wells were again washed 3x with DPBS. U937 cells were lysed by treatment with 1% saponin for 10 minutes and the number of viable intracellular bacteria was determined by serial dilution and plating of the lysate. Intracellular bacteria are reported as the fraction of total viable bacteria in the inoculum. Control wells containing bacteria only were also subjected to gentamicin treatment to test for complete bacterial killing following antibiotic exposure.

### Statistics

Uronic acid, sialic acid, serum killing, and U937 internalization measurements were conducted on three biological replicates and the results shown are representative of at least three independent experiments. Statistical analyses were conducted using Prism v.8 (GraphPad Software) and significance (alpha = 0.05) was calculated using an unpaired *t*-test or one-way ANOVA with Dunnett’s multiple comparisons test, as indicated. SDS-PAGE results are representative of at least two independent experiments.

## Supporting information

S1 Data *S*. *marcescens* KL identified in this study. (XLSX)

S1 Fig Mucoid colony morphology of KL2 strain gn773. (A) *S*. *marcescens* strain gn773 cultured on LB agar supplemented with glucose. (B) Qualitative demonstration of gn773 mucoidy by string test.
(EPS)

S2 Fig Detection of KDN in UMH9 and gn773 polysaccharide preparations. HPAEC-PAD was used to detect KDN from 5.6 μg of CPS isolated from strains UMH9 (A) and gn773 (B). Purified KDN (C) (1 nmole) was included as a control. The amount of KDN detected in each sample (nmole) is indicated in parentheses.
(EPS)

S3 Fig Sensitivity of wild-type and acapsular *S*. *marcescens* strains to heat-inactivated human serum. Bacteria were exposed to 40% heat-inactivated human serum for 90 minutes. Viability was calculated relative to the number of bacteria present at the start of the experiment. None of the tested strains differed significantly in sensitivity from the wild-type strain UMH9 as assessed by unpaired *t*-test (P>0.05).
(EPS)

S1 Table HPAEC-PAD monosaccharide composition of polysaccharides isolated from *S*. *marcescens* strains. (DOCX)

S2 Table Oligonucleotide primers used in this study. (DOCX)

## References

[1] European Centre for Disease Prevention and Control. Healthcare-associated infections acquired in intensive care units. Annual Epidemiological report for 2017. 2019. Available from: https://www.ecdc.europa.eu/sites/default/files/documents/AER_for_2017-HAI.pdf

[2] Wisplinghoff H, Bischoff T, Tallent SM, Seifert H, Wenzel RP, Edmond MB. Nosocomial bloodstream infections in US hospitals: analysis of 24,179 cases from a prospective nationwide surveillance study. Clin Infect Dis. 2004;39(3):309–17. doi: 10.1086/421946 15306996

[3] Diekema DJ, Hsueh PR, Mendes RE, Pfaller MA, Rolston KV, Sader HS, et al. The microbiology of bloodstream infection: 20-year trends from the SENTRY antimicrobial surveillance program. Antimicrob Agents Chemother. 2019;63(7): e00355–19. doi: 10.1128/AAC.00355-19 31010862 PMC6591610

[4] Johnson A, Watson D, Dreyfus J, Heaton P, Lampland A, Spaulding AB. Epidemiology of *Serratia* bloodstream infections among hospitalized children in the United States, 2009–2016. Pediatr Infect Dis J. 2020;39(6):e71–e3. doi: 10.1097/INF.0000000000002618 32091494

[5] Mahlen SD. *Serratia* infections: from military experiments to current practice. Clin Microbiol Rev. 2011;24(4):755–91. doi: 10.1128/CMR.00017-11 21976608 PMC3194826

[6] Grimont PA, Grimont F. Biotyping of *Serratia marcescens* and its use in epidemiological studies. J Clin Microbiol. 1978;8(1):73–83. doi: 10.1128/jcm.8.1.73-83.1978 353073 PMC275117

[7] Raymann K, Coon KL, Shaffer Z, Salisbury S, Moran NA. Pathogenicity of *Serratia marcescens* strains in honey bees. mBio. 2018;9(5):e01649–18. doi: 10.1128/mBio.01649-18 30301854 PMC6178626

[8] Patterson KL, Porter JW, Ritchie KB, Polson SW, Mueller E, Peters EC, et al. The etiology of white pox, a lethal disease of the Caribbean elkhorn coral, *Acropora palmata*. Proc Natl Acad Sci U S A. 2002;99(13):8725–30. doi: 10.1073/pnas.092260099 12077296 PMC124366

[9] Iguchi A, Nagaya Y, Pradel E, Ooka T, Ogura Y, Katsura K, et al. Genome evolution and plasticity of *Serratia marcescens*, an important multidrug-resistant nosocomial pathogen. Genome Biol Evol. 2014;6(8):2096–110. doi: 10.1093/gbe/evu160 25070509 PMC4231636

[10] Matteoli FP, Passarelli-Araujo H, Reis RJA, da Rocha LO, de Souza EM, Aravind L, et al. Genome sequencing and assessment of plant growth-promoting properties of a *Serratia marcescens* strain isolated from vermicompost. BMC Genomics. 2018;19(1):750. doi: 10.1186/s12864-018-5130-y 30326830 PMC6192313

[11] Moradigaravand D, Boinett CJ, Martin V, Peacock SJ, Parkhill J. Recent independent emergence of multiple multidrug-resistant *Serratia marcescens* clones within the United Kingdom and Ireland. Genome Res. 2016;26(8):1101–9. doi: 10.1101/gr.205245.116 27432456 PMC4971767

[12] Abreo E, Altier N. Pangenome of *Serratia marcescens* strains from nosocomial and environmental origins reveals different populations and the links between them. Sci Rep. 2019;9(1):46. doi: 10.1038/s41598-018-37118-0 30631083 PMC6328595

[13] Hume EB, Conerly LL, Moreau JM, Cannon BM, Engel LS, Stroman DW, et al. *Serratia marcescens* keratitis: strain-specific corneal pathogenesis in rabbits. Curr Eye Res. 1999;19(6):525–32. doi: 10.1076/ceyr.19.6.525.5283 10550795

[14] Aucken HM, Pitt TL. Different O and K serotype distributions among clinical and environmental strains of *Serratia marcescens*. J Med Microbiol. 1998;47(12):1097–104. doi: 10.1099/00222615-47-12-1097 9856646

[15] Franczek SP, Williams RP, Hull SI. A survey of potential virulence factors in clinical and environmental isolates of *Serratia marcescens*. J Med Microbiol. 1986;22(2):151–6. doi: 10.1099/00222615-22-2-151 3528499

[16] Anderson MT, Mitchell LA, Zhao L, Mobley HLT. Capsule production and glucose metabolism dictate fitness during *Serratia marcescens* bacteremia. mBio. 2017;8(3): e00740–17. doi: 10.1128/mBio.00740-17 28536292 PMC5442460

[17] Aucken HM, Wilkinson SG, Pitt TL. Identification of capsular antigens in *Serratia marcescens*. J Clin Microbiol. 1997;35(1):59–63. doi: 10.1128/jcm.35.1.59-63.1997 8968881 PMC229512

[18] Aucken HM, Wilkinson SG, Pitt TL. Re-evaluation of the serotypes of *Serratia marcescens* and separation into two schemes based on lipopolysaccharide (O) and capsular polysaccharide (K) antigens. Microbiology. 1998;144 (Pt 3)(3):639–53. 9534235 10.1099/00221287-144-3-639

[19] Whitfield C. Biosynthesis and assembly of capsular polysaccharides in *Escherichia coli*. Annu Rev Biochem. 2006;75(1):39–68. doi: 10.1146/annurev.biochem.75.103004.142545 16756484

[20] Pan YJ, Lin TL, Chen CT, Chen YY, Hsieh PF, Hsu CR, et al. Genetic analysis of capsular polysaccharide synthesis gene clusters in 79 capsular types of *Klebsiella* spp. Sci Rep. 2015;5:15573. doi: 10.1038/srep15573 26493302 PMC4616057

[21] Whitfield C, Wear SS, Sande C. Assembly of bacterial capsular polysaccharides and exopolysaccharides. Annu Rev Microbiol. 2020;74:521–43. doi: 10.1146/annurev-micro-011420-075607 32680453

[22] Cuthbertson L, Mainprize IL, Naismith JH, Whitfield C. Pivotal roles of the outer membrane polysaccharide export and polysaccharide copolymerase protein families in export of extracellular polysaccharides in Gram-negative bacteria. Microbiol Mol Biol Rev. 2009;73(1):155–77. doi: 10.1128/MMBR.00024-08 19258536 PMC2650888

[23] Goh KGK, Phan MD, Forde BM, Chong TM, Yin WF, Chan KG, et al. Genome-wide discovery of genes required for capsule production by uropathogenic *Escherichia coli*. mBio. 2017;8(5): e01558–17. doi: 10.1128/mBio.01558-17 29066548 PMC5654933

[24] Woodward R, Yi W, Li L, Zhao G, Eguchi H, Sridhar PR, et al. *In vitro* bacterial polysaccharide biosynthesis: defining the functions of Wzy and Wzz. Nat Chem Biol. 2010;6(6):418–23. doi: 10.1038/nchembio.351 20418877 PMC2921718

[25] Kawai E, Akatsuka H, Idei A, Shibatani T, Omori K. *Serratia marcescens* S-layer protein is secreted extracellularly via an ATP-binding cassette exporter, the Lip system. Mol Microbiol. 1998;27(5):941–52. doi: 10.1046/j.1365-2958.1998.00739.x 9535084

[26] Kurz CL, Chauvet S, Andres E, Aurouze M, Vallet I, Michel GP, et al. Virulence factors of the human opportunistic pathogen *Serratia marcescens* identified by *in vivo* screening. EMBO J. 2003;22(7):1451–60. doi: 10.1093/emboj/cdg159 12660152 PMC152903

[27] Romanowski EG, Stella NA, Romanowski JE, Yates KA, Dhaliwal DK, St Leger AJ, et al. The Rcs stress response system regulator GumB modulates *Serratia marcescens*-induced inflammation and bacterial proliferation in a rabbit keratitis model and cytotoxicity *in vitro*. Infect Immun. 2021;89(8):e0011121. doi: 10.1128/IAI.00111-21 33820815 PMC8281226

[28] Rahn A, Beis K, Naismith JH, Whitfield C. A novel outer membrane protein, Wzi, is involved in surface assembly of the *Escherichia coli* K30 group 1 capsule. J Bacteriol. 2003;185(19):5882–90. doi: 10.1128/JB.185.19.5882-5890.2003 13129961 PMC193962

[29] Bushell SR, Mainprize IL, Wear MA, Lou H, Whitfield C, Naismith JH. Wzi is an outer membrane lectin that underpins group 1 capsule assembly in *Escherichia coli*. Structure. 2013;21(5):844–53. doi: 10.1016/j.str.2013.03.010 23623732 PMC3791409

[30] Annunziato PW, Wright LF, Vann WF, Silver RP. Nucleotide sequence and genetic analysis of the *neuD* and *neuB* genes in region 2 of the polysialic acid gene cluster of *Escherichia coli* K1. J Bacteriol. 1995;177(2):312–9. doi: 10.1128/jb.177.2.312-319.1995 7814319 PMC176593

[31] Vann WF, Tavarez JJ, Crowley J, Vimr E, Silver RP. Purification and characterization of the *Escherichia coli* K1 *neuB* gene product *N*-acetylneuraminic acid synthetase. Glycobiology. 1997;7(5):697–701. doi: 10.1093/glycob/7.5.697 9254051

[32] Warren L. The thiobarbituric acid assay of sialic acids. J Biol Chem. 1959;234(8):1971–5. 13672998

[33] Vimr ER, Kalivoda KA, Deszo EL, Steenbergen SM. Diversity of microbial sialic acid metabolism. Microbiol Mol Biol Rev. 2004;68(1):132–153. doi: 10.1128/MMBR.68.1.132-153.2004 15007099 PMC362108

[34] Vinogradov E, Petersen BO, Duus JO, Radziejewska-Lebrecht J. The structure of the polysaccharide part of the LPS from *Serratia marcescens* serotype O19, including linkage region to the core and the residue at the non-reducing end. Carbohydr Res. 2003;338(23):2757–61. doi: 10.1016/j.carres.2003.08.011 14670734

[35] Coderch N, Pique N, Lindner B, Abitiu N, Merino S, Izquierdo L, et al. Genetic and structural characterization of the core region of the lipopolysaccharide from *Serratia marcescens* N28b (serovar O4). J Bacteriol. 2004;186(4):978–88. doi: 10.1128/JB.186.4.978-988.2004 14761992 PMC344232

[36] Mostowy RJ, Holt KE. Diversity-generating machines: genetics of bacterial sugar-coating. Trends Microbiol. 2018;26(12):1008–21. doi: 10.1016/j.tim.2018.06.006 30037568 PMC6249986

[37] Holt KE, Lassalle F, Wyres KL, Wick R, Mostowy RJ. Diversity and evolution of surface polysaccharide synthesis loci in *Enterobacteriales*. ISME J. 2020;14(7):1713–30. doi: 10.1038/s41396-020-0628-0 32249276 PMC7305143

[38] Tickner J, Hawas S, Totsika M, Kenyon JJ. The Wzi outer membrane protein mediates assembly of a tight capsular polysaccharide layer on the *Acinetobacter baumannii* cell surface. Sci Rep. 2021;11(1):21741. doi: 10.1038/s41598-021-01206-5 34741090 PMC8571296

[39] Lawrence SM, Huddleston KA, Pitts LR, Nguyen N, Lee YC, Vann WF, et al. Cloning and expression of the human *N*-acetylneuraminic acid phosphate synthase gene with 2-keto-3-deoxy-D-glycero- D-galacto-nononic acid biosynthetic ability. J Biol Chem. 2000;275(23):17869–77. doi: 10.1074/jbc.M000217200 10749855

[40] Gulati S, Schoenhofen IC, Lindhout-Djukic T, Schur MJ, Landig CS, Saha S, et al. Therapeutic CMP-nonulosonates against multidrug-resistant *Neisseria gonorrhoeae*. J Immunol. 2020;204(12):3283–95. doi: 10.4049/jimmunol.1901398 32434942 PMC7321800

[41] Gulati S, Schoenhofen IC, Whitfield DM, Cox AD, Li J, St Michael F, et al. Utilizing CMP-sialic acid analogs to unravel *Neisseria gonorrhoeae* lipooligosaccharide-mediated complement resistance and design novel therapeutics. PLoS Pathog. 2015;11(12):e1005290. doi: 10.1371/journal.ppat.1005290 26630657 PMC4668040

[42] Saha S, Coady A, Sasmal A, Kawanishi K, Choudhury B, Yu H, et al. Exploring the impact of ketodeoxynonulosonic acid in host-pathogen interactions using uptake and surface display by nontypeable *Haemophilus influenzae*. mBio. 2021;12(1): e03226–20. doi: 10.1128/mBio.03226-20 33468699 PMC7845648

[43] Knirel YA, Kocharova NA, Shashkov AS, Kochetkov NK, Mamontova VA, Soloveva TF. Structure of the capsular polysaccharide of *Klebsiella ozaenae* serotype K4 containing 3-deoxy-D-glycero-D-galacto-nonulosonic acid. Carbohydr Res. 1989;188:145–55. doi: 10.1016/0008-6215(89)84067-4 2776127

[44] Gil-Serrano AM, Rodriguez-Carvajal MA, Tejero-Mateo P, Espartero JL, Thomas-Oates J, Ruiz-Sainz JE, et al. Structural determination of a 5-O-methyl-deaminated neuraminic acid (Kdn)-containing polysaccharide isolated from *Sinorhizobium fredii*. Biochem J. 1998;334 (Pt 3):585–94. doi: 10.1042/bj3340585 9729466 PMC1219727

[45] Wang L, Lu Z, Allen KN, Mariano PS, Dunaway-Mariano D. Human symbiont *Bacteroides thetaiotaomicron* synthesizes 2-keto-3-deoxy-D-glycero-D-galacto-nononic acid (KDN). Chem Biol. 2008;15(9):893–7. doi: 10.1016/j.chembiol.2008.08.005 18804026 PMC4425688

[46] Xu J, Mahowald MA, Ley RE, Lozupone CA, Hamady M, Martens EC, et al. Evolution of symbiotic bacteria in the distal human intestine. PLoS Biol. 2007;5(7):e156. doi: 10.1371/journal.pbio.0050156 17579514 PMC1892571

[47] Shashkov AS, Kosmachevskaya LN, Streshinskaya GM, Evtushenko LI, Bueva OV, Denisenko VA, et al. A polymer with a backbone of 3-deoxy-D-glycero-D-galacto-non-2-ulopyranosonic acid, a teichuronic acid, and a beta-glucosylated ribitol teichoic acid in the cell wall of plant pathogenic *Streptomyces* sp. VKM Ac-2124. Eur J Biochem. 2002;269(24):6020–5. doi: 10.1046/j.1432-1033.2002.03274.x 12473097

[48] Shashkov AS, Tul’skaya EM, Evtushenko LI, Denisenko VA, Ivanyuk VG, Stomakhin AA, et al. Cell wall anionic polymers of *Streptomyces* sp. MB-8, the causative agent of potato scab. Carbohydr Res. 2002;337(21–23):2255–61. doi: 10.1016/s0008-6215(02)00188-x 12433490

[49] Inoue S, Lin SL, Chang T, Wu SH, Yao CW, Chu TY, et al. Identification of free deaminated sialic acid (2-keto-3-deoxy-D-glycero-D-galacto-nononic acid) in human red blood cells and its elevated expression in fetal cord red blood cells and ovarian cancer cells. J Biol Chem. 1998;273(42):27199–204. doi: 10.1074/jbc.273.42.27199 9765240

[50] Angata T, Varki A. Chemical diversity in the sialic acids and related alpha-keto acids: An evolutionary perspective. Chem Rev. 2002;102(2):439–69. doi: 10.1021/cr000407m 11841250

[51] Paoletti LC, Kasper DL. Surface structures of group B *Streptococcus* important in human immunity. Microbiol Spectr. 2019;7(2). doi: 10.1128/microbiolspec.GPP3-0001-2017 30873933 PMC11590616

[52] Barry GT. Colominic acid, a polymer of *N*-acetylneuraminic acid. J Exp Med. 1958;107(4):507–21. doi: 10.1084/jem.107.4.507 13513915 PMC2136837

[53] Bhattacharjee AK, Jennings HJ, Kenny CP, Martin A, Smith IC. Structural determination of the sialic acid polysaccharide antigens of *Neisseria meningitidis* serogroups B and C with carbon 13 nuclear magnetic resonance. J Biol Chem. 1975;250(5):1926–32. 163259

[54] Mandrell RE, Apicella MA. Lipo-oligosaccharides (LOS) of mucosal pathogens: molecular mimicry and host-modification of LOS. Immunobiology. 1993;187(3–5):382–402. doi: 10.1016/S0171-2985(11)80352-9 8330904

[55] Carlin AF, Uchiyama S, Chang YC, Lewis AL, Nizet V, Varki A. Molecular mimicry of host sialylated glycans allows a bacterial pathogen to engage neutrophil Siglec-9 and dampen the innate immune response. Blood. 2009;113(14):3333–6. doi: 10.1182/blood-2008-11-187302 19196661 PMC2665898

[56] Chang YC, Olson J, Beasley FC, Tung C, Zhang J, Crocker PR, et al. Group B *Streptococcus* engages an inhibitory Siglec through sialic acid mimicry to blunt innate immune and inflammatory responses *in vivo*. PLoS Pathog. 2014;10(1):e1003846. doi: 10.1371/journal.ppat.1003846 24391502 PMC3879367

[57] Simon R, Priefer U, Puhler A. A broad host range mobilization system for *in vivo* genetic-engineering—transposon mutagenesis in Gram-negative bacteria. Bio-Technol. 1983;1(9):784–91.

[58] Bertani G. Studies on lysogenesis. I. The mode of phage liberation by lysogenic *Escherichia coli*. J Bacteriol. 1951;62(3):293–300. doi: 10.1128/jb.62.3.293-300.1951 14888646 PMC386127

[59] Miller JH. Experiments in molecular genetics. Cold Spring Harbor, N. Y.: Cold Spring Harbor Laboratory; 1972.

[60] Datsenko KA, Wanner BL. One-step inactivation of chromosomal genes in *Escherichia coli* K-12 using PCR products. Proc Natl Acad Sci U S A. 2000;97(12):6640–5. doi: 10.1073/pnas.120163297 10829079 PMC18686

[61] Datta S, Costantino N, Court DL. A set of recombineering plasmids for Gram-negative bacteria. Gene. 2006;379:109–15. doi: 10.1016/j.gene.2006.04.018 16750601

[62] Thomason LC, Sawitzke JA, Li X, Costantino N, Court DL. Recombineering: genetic engineering in bacteria using homologous recombination. Curr Protoc Mol Biol. 2014;106:1.16.1–39. doi: 10.1002/0471142727.mb0116s106 24733238

[63] Lazarus JE, Warr AR, Kuehl CJ, Giorgio RT, Davis BM, Waldor MK. A new suite of allelic-exchange vectors for the scarless modification of proteobacterial genomes. Appl Environ Microbiol. 2019;85(16):e00990–19. doi: 10.1128/AEM.00990-19 31201277 PMC6677854

[64] Ferrieres L, Hemery G, Nham T, Guerout AM, Mazel D, Beloin C, et al. Silent mischief: bacteriophage Mu insertions contaminate products of *Escherichia coli* random mutagenesis performed using suicidal transposon delivery plasmids mobilized by broad-host-range RP4 conjugative machinery. J Bacteriol. 2010;192(24):6418–27. doi: 10.1128/JB.00621-10 20935093 PMC3008518

[65] Katoh K, Rozewicki J, Yamada KD. MAFFT online service: multiple sequence alignment, interactive sequence choice and visualization. Brief Bioinform. 2017. 10.1093/bib/bbx108 PMC6781576 28968734

[66] Letunic I, Bork P. Interactive tree of life (iTOL) v5: an online tool for phylogenetic tree display and annotation. Nucleic Acids Res. 2021;49(W1):W293–W6. doi: 10.1093/nar/gkab301 33885785 PMC8265157

[67] Favre-Bonte S, Joly B, Forestier C. Consequences of reduction of *Klebsiella pneumoniae* capsule expression on interactions of this bacterium with epithelial cells. Infect Immun. 1999;67(2):554–61. doi: 10.1128/IAI.67.2.554-561.1999 9916058 PMC96354

[68] Blumenkrantz N, Asboe-Hansen G. New method for quantitative determination of uronic acids. Anal Biochem. 1973;54(2):484–9. doi: 10.1016/0003-2697(73)90377-1 4269305

[69] Domenico P, Diedrich DL, Cunha BA. Quantitative extraction and purification of exopolysaccharides from *Klebsiella pneumoniae*. J Microbiol Methods. 1989;9(3):211–9.

[70] Tipton KA, Rather PN. Extraction and visualization of capsular polysaccharide from *Acinetobacter baumannii*. Methods Mol Biol. 2019;1946:227–31. doi: 10.1007/978-1-4939-9118-1_21 30798559

