# Supplementary material for: Identification of distinct capsule types associated with Serratia marcescens infection isolates
Source: PLoS Pathog. 2022 Mar 30;18(3):e1010423. doi: 10.1371/journal.ppat.1010423 (PMC9000132; doi:10.1371/journal.ppat.1010423)
Supplement: S1 Table — (DOCX) [file ppat.1010423.s005.docx]

**S1 Table. HPAEC-PAD monosaccharide composition of polysaccharides isolated from *S. marcescens* strains.**

| **Monosaccharide**^a^ | **Relative peak area (%)** | | | | | |
| --- | --- | --- | --- | --- | --- | --- |
|  | **UMH9** | **UMH9 ΔCPS_v_::*nptII*** | **gn773** | **UMH7** | **UMH11** | **ATCC 13880** |
| Gal | -^b^ | - | 28.69 | - | - | - |
| GalNH_2_ | 15.99 | 31.73 | - | - | 24.20 | 0.41 |
| Glc | 42.55 | 17.36 | 37.78 | 27.93 | 20.87 | 32.84 |
| GlcA | 9.09 | - | 14.36 | 11.71 | 6.58 | - |
| GlcNH_2_ | 3.38 | 8.64 | 1.92 | 23.20 | 5.02 | 11.17 |
| Hep | 3.59 | 8.01 | - | 1.00 | 6.34 | - |
| Man | 9.01 | - | 15.79 | 28.75 | 14.43 | - |
| Rha | 1.30 | 2.88 | - | - | 2.43 | - |
| Rib | 15.09 | 29.68 | - | 7.41 | 20.14 | 51.85 |
| Xyl | - | 1.71 | 1.46 | - | - | 3.49 |

^a^, abbreviations: Gal, galactose; GalNH_2_, galactosamine; Glc, glucose; GlcA, glucuronic acid; GlcNH_2_, glucosamine; Hep, heptose; Man, mannose; Rha, rhamnose; Rib, ribose; Xyl, xylose

^b^, not quantified
