# Supplementary material for: Identification of distinct capsule types associated with Serratia marcescens infection isolates
Source: PLoS Pathog. 2022 Mar 30;18(3):e1010423. doi: 10.1371/journal.ppat.1010423 (PMC9000132; doi:10.1371/journal.ppat.1010423)
Supplement: S2 Table — (DOCX) [file ppat.1010423.s006.docx]

**S2 Table. Oligonucleotide primers used in this study.**

|  | | |
| --- | --- | --- |
| **Name** | **Sequence (5ʹ-3ʹ)** | **Description** |
| SDH_P1 | NNNNtctagaccggtaatcgtcttggtggc | *neuB*-containing fragment with 128-bp of upstream and 133-bp of downstream sequence |
| SDH_P2 | NNNNctcgagaataaagagtcagcgcgccg |  |
| SDH_P3 | gccttcttgacgagttcttctaaatgagtaaagccattat | generation of *neuB* deletion allele in pCR2.1 |
| SDH_P4 | tgcaatccatcttgttcaatcataaattatctcccactcc |  |
| SDH_P5 | ggagtgggagataatttatgattgaacaagatggattgca | *nptII* amplicon for insertion into the *neuB* deletion allele |
| SDH_P6 | ataatggctttactcatttagaagaactcgtcaagaaggc |  |
| SDH_P7 | gagagggtaccgcatgagcttggcgattgaagccgtcaga | fragment containing first 30-bp of *neuA* with pTOX11*_nptII_* complementarity for generation of Δ*neuAB* allele |
| SDH_P8 | ttttccaggaagttaagcgcccttgccggaataattgcaa |  |
| SDH_P9 | ttgcaattattccggcaagggcgcttaacttcctggaaaa | fragment containing last 30-bp of *neuB* with pTOX11*_nptII_* complementarity for generation of Δ*neuAB* allele |
| SDH_P10 | caagaaaacaggacacttggcatgtaatgcgctatgaaga |  |
| SDH_P11 | cagctatgaccatgattacgagataatttatgttaattca | *neuB* linear fragment with pBBR1MCS-5 complementarity |
| SDH_P12 | agcgcgcgtaatacgactcaactcatttacagatactttt |  |
| SDH_P13 | tgagtcgtattacgcgcgctcactg | pBBR1MCS-5 backbone amplicon |
| SDH_P14 | cgtaatcatggtcatagctgtttcc |  |
| SDH_P15 | caactctctactgtttctccctacggctactacgcttacg | *neuAB* fragment with 119-bp upstream and 67-bp downstream sequence |
| SDH_P16 | atttaatctgtatcaggctgagccgatgaccttgccgaga |  |
| SDH_P17 | cagcctgatacagattaaat | pBAD18-kan backbone amplicon |
| SDH_P18 | ggagaaacagtagagagttg |  |
| MTA_P1 | ttctgattcattcaaaagtaatgcataaaatccaatttcggagagaataatgtaggctggagctgcttcg | ΔCPSv::*nptII* linear fragment |
| MTA_P2 | ctaccagggcgaggcaatcggagcactggagcgcatcattaccggtacatcatatgaatatcctccttagt |  |
| MTA_P3 | ctttacataaaaccaataacctcgaattaacaagggaattctttatgcgctgtaggctggagctgcttcg | Δ*wzi*::*nptII* linear fragment |
| MTA_P4 | acgtctgcaaaaaattacaggttgatcggcacttcgaagctgacgccggccatatgaatatcctccttagt |  |
| MTA_P5 | gttcaatcatatgtttttcctccttatgttaagc | pTOX11 amplicon lacking *aacC1* gene |
| MTA_P6 | gttcttctgaagatccttggcggcaagaaaac |  |
| MTA_P7 | cataaggaggaaaaacatatgattgaacaagatggattg | *nptII* linear fragment with pTOX11 complementarity |
| MTA_P8 | ttttcttgccgccaaggatcttcagaagaactcgtcaagaag |  |
| MTA_P9 | agctcatgcggtaccctctc | pTOX11*_nptII_* backbone amplicon |
| MTA_P10 | ccaagtgtcctgttttcttg |  |
| MTA_P11 | gcgcggtacccaactatcatgggacacagaga | promoterless *wzi* fragment |
| MTA_P12 | gcgcaagctttggcgggcagcgagaaaatcag |  |
